# The development of a theory informed behaviour change intervention to improve adherence to dietary and physical activity treatment guidelines in individuals with familial hypercholesterolaemia (FH)

**DOI:** 10.1186/s12913-019-4869-4

**Published:** 2020-01-08

**Authors:** F. J. Kinnear, E. Wainwright, J. E. Bourne, F. E. Lithander, J. Hamilton-Shield, A. Searle

**Affiliations:** 1 NIHR Bristol Biomedical Research Centre (Nutrition Theme), University Hospitals Bristol NHS Foundation Trust and the University of Bristol, Bristol, UK; 2 Psychology Department, Bath Spa University and Honorary Research Fellow, Department for Health, University of Bath, Bath, UK

## Abstract

**Background:**

Familial hypercholesterolaemia (FH) is a genetic condition characterised by elevated levels of low-density lipoprotein cholesterol (LDL-C) and an increased risk of cardiovascular disease (CVD). Following dietary and physical activity guidelines could help minimise this risk but adherence is low. Interventions to target these behaviours are therefore required. A comprehensive understanding of the target behaviours and behaviour change theory should drive the process of intervention development to increase intervention effectiveness and scalability. This paper describes the application of a theoretical framework to the findings of a qualitative evidence synthesis (QES) to inform the content and delivery of an intervention to improve adherence to dietary and physical activity guidelines in individuals with FH.

**Methods:**

The Behaviour Change Wheel (BCW) was used to guide intervention development. Factors influencing dietary and physical activity behaviours were identified from an earlier QES and mapped onto factors within the BCW. A comprehensive behavioural diagnosis of these factors was conducted through application of the theoretical domains framework (TDF). Using these data, the most appropriate intervention functions and behaviour change techniques (BCTs) for inclusion in the intervention were identified. Decision making was guided by evaluation criteria recommended by BCW guidance and feedback from individuals with FH.

**Results:**

Factors influencing dietary and physical activity behaviours mapped onto twelve of the fourteen TDF domains, with seven intervention functions deemed suitable to target the domains’ theoretical constructs. Twenty-six BCTs were identified as being appropriate for delivery within these functions and were included in the intervention. For instance, within the *enablement* intervention function, the BCT *problem solving* was incorporated by inclusion of a ‘barriers and solutions’ section. Guided by evaluation criteria and feedback from individuals with FH, the intervention will be delivered as an hour-long family-based appointment, followed up with four telephone calls.

**Conclusions:**

The novel application of the BCW and TDF to the results of a QES has enabled the development of a theory and evidence informed behaviour change intervention. This systematic approach facilitates evaluation of the intervention as part of an ongoing feasibility trial. The transparent approach taken can be used to guide intervention development by researchers in other fields.

## Background

Heterozygous familial hypercholesterolaemia (FH) is a genetic disorder characterised by elevated levels of low-density lipoprotein cholesterol (LDL-C) [1] leading to a substantially increased risk of cardiovascular disease (CVD) [2]. Despite pharmacological treatment, many affected individuals remain at a higher CVD risk than the general population [2–4]. Affecting 1 in 250 individuals worldwide [5], this excess CVD risk is a recognised public health concern [6]. Non-attainment of LDL-C treatment targets and the presence of other CVD risk factors account for a substantial proportion of this excess risk [7–9]. In light of the proven benefits of a healthy diet and physical activity upon CVD risk factors in the wider population [10, 11], current FH management guidelines emphasise the importance of dietary and physical activity treatment guidelines [12, 13]. However adherence has been found to be sub-optimal [14–16] and given the potential positive impact of this component of treatment, effective interventions designed to improve these behaviours in individuals with FH are required. This paper describes the development of a theoretically informed behaviour change intervention to improve adherence to dietary and physical activity treatment guidelines in individuals with FH.

To develop an effective and scalable intervention, a comprehensive understanding of the target behaviours and the factors influencing them is required. The specific intervention content designed to bring about behaviour change (i.e. the active ingredients) should be driven by theory and take into account situational contexts in which the intervention will be delivered and received [17, 18]. Several aspects of FH, an inherited condition, contribute to a unique context that determines the extent to which these individuals with FH will engage in physical activity and dietary behaviours. These include its asymptomatic nature if compliant with treatment, comparisons with high cholesterol levels caused by lifestyle factors alone, lifelong treatment, outpatient management with infrequent contact with healthcare professionals and a genetic inheritance pattern resulting in each affected individual having at least one affected parent [16]. Due to these several interacting components and the multiple behaviours required to be targeted, the development of a complex intervention is required [17, 18]. While complex interventions are common within health research, it is often difficult to evaulate their effectiveness due to the presence of several, often interacting, components [19, 20]. This limits the ability to refine and improve interventions that are ineffective or replicate effective intervention in other contexts [19, 20]. To overcome this recognised problem in health research, The Medical Research Council (MRC) framework for the development and evaulation of complex interventions advises that intervention developers should apply a systematic and transparent approach when designing complex interventions [17].

In the guidance, the MRC recommends including theory in the development of complex interventions, however it does not specify which of the many theories of behaviour change should be used or how the most appropriate theoretical model should be selected [17, 18]. In the absence of a theoretical evaluation and understanding of the target behaviour(s) within the FH population to guide the selection of a theoretical model, any one theory chosen may not encompass all the relevant theoretical constructs required to bring about effective behaviour change and/or include unnecessary constructs [21]. The current lack of theoretical understanding of how having FH influences behaviours could explain why a previous behaviour change intervention to improve the dietary intake and physical activity levels of individuals with FH was unsuccessful [22, 23]. The intervention used in the trial was developed using the *I-Change* model of behavioural change [22, 23]. While this model has been successfully applied to understand sunscreen use in adolescents [24], its failure to elicit the desired changes in dietary and physical activity behaviours suggest it might not have been suitable to address the relevant theoretical constructs in an appropriate way for the context of FH. To date there are no other published evaluations of behaviour change interventions conducted in FH cohorts. Within cohorts of individuals with other chronic conditions, interventions have had limited success in improving lifestyle behaviours [25, 26]. Furthermore, previous interventions that targeted healthy eating and physical activity behaviours have rarely used theory and when they have, it was inadequately reported meaning the effectiveness of any theoretical model applied is hard to determine [27–29].

**Fig. 1:**
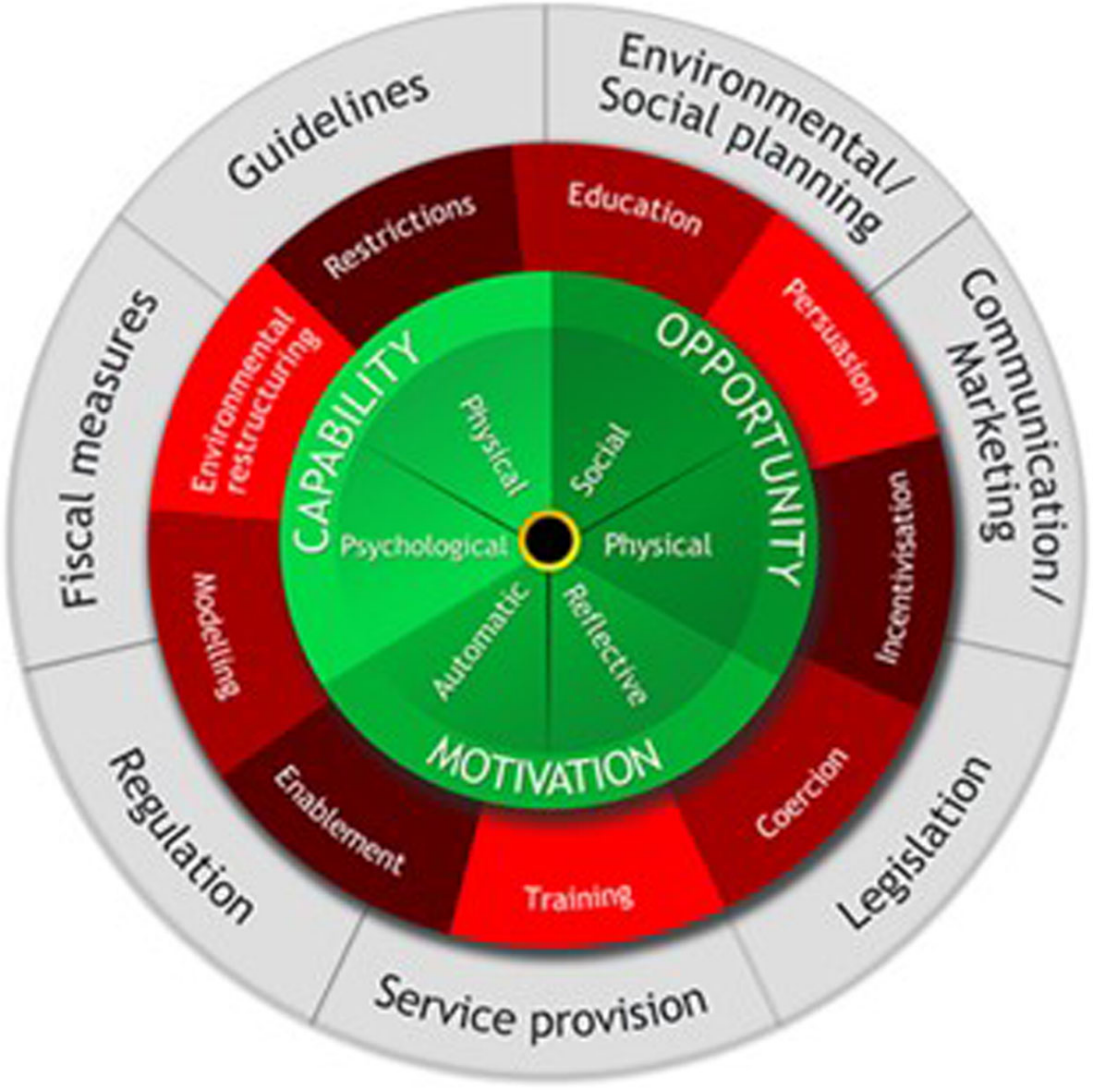
The Behaviour Change Wheel. The sources of behaviour are shown in green, the intervention functions in red and the policy categories in grey. Reproduced from Susan Michie et al. [30]

The Behaviour Change Wheel (BCW) (Fig. 1) was created in recognition of the fact that while there are many important drivers of behaviours, no single theoretical framework recognises them all [31]. The BCW integrates 19 behaviour change frameworks, linking them to a model which is broad enough to be applied to any type of behaviour across a variety of settings, negating the need to select one specific theoretical approach [31]. Utilising the BCW enables the subsequent identification of appropriate behaviour change techniques (BCTs) to target the theoretical constructs that may potentially influence the target behaviours [30]. The ‘COM-B model’ of behaviour upon which the BCW centres, depicts behaviours as being the result of interactions between ‘capability’, ‘opportunity’, and ‘motivation’. This model is intended to guide identification of the sources of behaviour to target in interventions. The second layer of the BCW displays the intervention functions to target the COM-B components (e.g. *education*) and the third layer comprises of policy categories that could be used to deliver interventions (e.g. *regulation*). The application of the BCW facilitates a systematic and explicit approach to the design and reporting of the development of a complex intervention, in line with MRC guidance [17]. This allows an in depth evaluation to understand how and why an intervention had an effect, which facilitates refinement if required or replication in other settings [20, 32, 33].

The application of the BCW as a framework to intepret primary qualitative research findings to inform intervention development and identify BCTs has been widely reported across a variety of settings [34–36]. However, to our knowledge it has not been used to design an intervention to target dietary or physical activity behaviours in children and adults with FH. Furthermore, published examples of intervention development processes that utilised the BCW approach upon qualitative evidence synthesis (QES) findings, as opposed to primary qualitative research, have rarely been described [37]. QES can overcome the limitations of single qualitative research studies, from which findings may have limited transferability outside the sample in which they were conducted [38], increasing the potential acceptability, effectiveness and scalability of the intervention developed [37]. Analysing the findings of the QES using the BCW produces a deeper theoretical understanding of the identified barriers and faciltators and the subsequent identification of appropriate BCTs [27].

This paper describes the development process of a complex behaviour change intervention which is currently being evaluated as part of a randomised controlled feasibility trial (ISRCTN24880714). This paper will describe how the BCW was used to provide theoretical context to the findings of a QES to a) inform the content of the intervention, including selection of appropriate BCTs and b) identify the most appropriate mode of delivery. This process can be adopted by other healthcare professionals or researchers who wish to design effective interventions combining both patient understanding of their disorder and theoretical guidance.

## Methods

### The feasibility trial

The intervention was developed for use in a research trial investigating the feasibility and acceptability of delivering an intervention aimed at reducing cardiovascular risk factors in individuals with FH through improving adherence to currently recommended dietary and physical activity treatment advice, described in full elsewhere (ISRCTN24880714). The trial aims to address the findings of recent systematic reviews which have highlighted the lack of evidence investigating the efficacy of providing this advice upon the management of CVD risk factors in this population group [39, 40]. Given the available research suggesting that adherence to dietary and physical advice is sub-optimal [14] and the lack of success of a previous intervention in individuals with FH [23, 41], a theory informed behaviour change intervention tailored to the specific drivers of behaviour within individuals with FH is required. The intervention development process described here was carried out prior to its subsequent utilisation in the feasibility trial, in which the acceptability and potential effectiveness of the intervention is to be evaluated.

### Overview of the BCW approach to intervention development

**Fig. 2:**
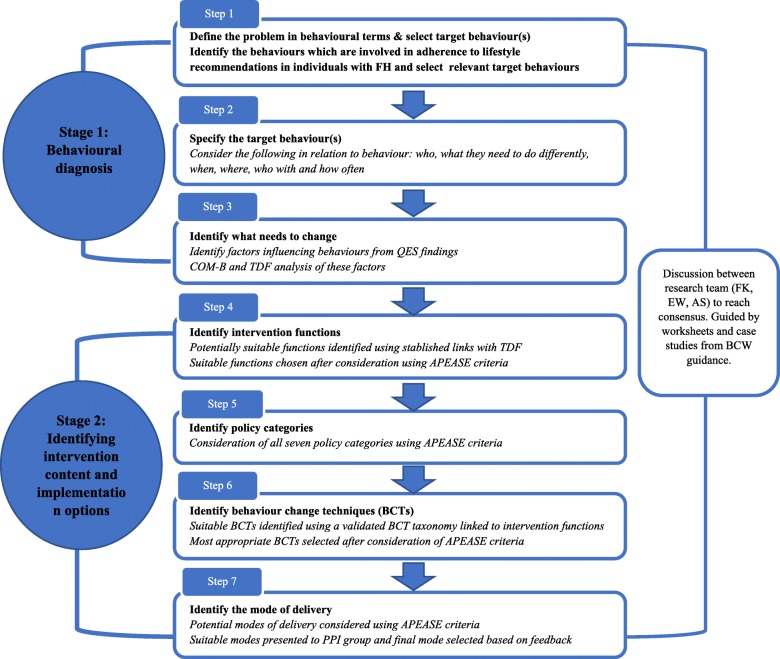
Flow diagram of the steps followed in the development of the intervention

Members of the research team (FJK, EW, AS) followed the recommended stages and steps of the widely used BCW approach [31] in the development of the intervention described below and displayed in Fig. 2. The approach involves seven steps which are best understood within 2 broad stages: 1) Behavioural diagnosis and 2) Identifying intervention content and implementation options. This step by step process was conducted through a series of team meetings which were structured by the guidance provided by the developers of the BCW approach [30]. This guidance provides worksheets and case studies to assist researchers in their translation of the findings at each step of the process. The research team applied the BCW recommended APEASE criteria (affordability, practicality, effectiveness and cost effectiveness, acceptability, side-effects/safety and equity) [42] to assist with decision making regarding intervention content and delivery method. This facilitated evaluation of the appropriateness and suitability of the identified functions, BCTs and intervention delivery modes within the context of the feasibility trial in which the intervention was to be delivered. For each step, the research team met to discuss their individual findings, before reaching a group consensus. Additional members of the research team involved in the feasibility trial (FEL, JHS, GB, DS, AT) also provided input and feedback throughout the development process.

### The qualitative evidence synthesis (QES)

In the development of this intervention the BCW approach was applied to the results of a previously conducted qualitative evidence synthesis (QES) [16, 43]. The aim of this QES was to identify the enablers and barriers faced by individuals with FH in relation to adhering to lifestyle (dietary and physical activity guidelines) and pharmacological treatment. The results were translated into pragmatic recommendations for clinical practice i.e. *Treatment advice to be provided in family-based clinics*. For the purpose of intervention development, in this current approach, the identified enablers and barriers were examined in more depth using the BCW approach. This allowed for exploration of the theoretical determinants of the identified enablers and barriers and identification of the individual and environmental changes required to bring about change in the target behaviours. Members of the current research team (FJK, EW, AS) also undertook the QES analysis and were therefore familiar with the primary data included in the synthesis and the descriptive and thematic themes generated. For further details of the methodological approach taken when carrying out the QES and a richer description of themes uncovered, readers are directed to the published protocol and results [16, 43].

### Stage 1: Behavioural diagnosis

The first two steps within this stage aim to help the research team to define the behavioural problem(s) that the intervention aims to change before selecting and detailing the specific behaviour(s) to target. The third step involves a behavioural analysis to identify what needs to change to bring about the desired change in the target behaviour(s). These could be factors that need to change within the person and/or their environment.

#### Step 1: define the problem in behavioural terms & select target behaviour(s)

The research team reviewed and discussed the QES findings relating to the problem that the intervention is being designed to address: poor adherence to the dietary and physical activity guidelines for individuals with FH. Specifically, the research team considered the behaviours involved with dietary choices to achieve the current recommended dietary and physical activity guidelines for individuals with FH which are [10, 13, 44]:

1. Total daily fat intake ≤30% of total energy intake (TEI)
2. Daily saturated fat intake of ≤10% TEI achieved via replacement of saturated fats with monounsaturated and polyunsaturated fats
3. Daily dietary cholesterol intake ≤300 mg
4. Consumption of ≥5 portions of fruit and vegetables a day
5. Age appropriate fibre intake: 10-year-olds = 20 g/day; 11–16 year-olds = 25 g/day and 30 g/day for ≥17 years
6. 2 g of plant stanol/sterols per day
7. Reduce time spent engaged in sedentary behaviors
8. Age appropriate physical activity levels:

- Adults aged 19–64 years: ≥ 150 min a week of moderate intensity physical activity or ≥ 75 min of vigorous intensity physical activity, or a mixture of the two. Additional activity focussing on improving muscle strength should be undertaken twice a week.
- Young people aged 5–18 years: ≥ 60 min of moderate-to-vigorous physical activity each day, with 3 of these sessions each week being of vigorous intensity and including activities that strengthen muscle and bone.

#### Step 2: specify the target behaviour(s)

To gain a deeper understanding of the target behaviours the research team considered and discussed the following aspects in relation to the target behaviour: which individuals need to engage in the behaviour; what they need to do differently to enable the behaviour change; when, where, who with and how often they will engage in the behaviours.

#### Step 3: identify what needs to change

This step involves identifying what needs to change in the individual and/or the environment in which they live to bring about the desired changed in behaviour. This was achieved by carrying out a COM-B analysis based upon the findings of the QES. In this analysis, each component of the COM-B model of behaviour change (shown in green in Fig. 1) is considered in relation to the target behaviour. To facilitate a deeper understanding of the behaviour, the research team carried out a further behavioural analysis on the COM-B components identified as relevant. This was carried out using the Theoretical Domains Framework (TDF), which is an extension of the COM-B model. The TDF is comprised of 14 theoretical domains (e.g. *behavioural regulation*) which summarise the theoretical constructs from 33 theories of behaviour change [21]. The 14 domains have been linked to the COM-B model components (Table 2) and provide a framework for a more comprehensive behavioural diagnosis to identify the drivers of behaviours and to guide the subsequent identification of suitable intervention functions that can be used to promote change in these behaviours. The TDF has been validated for use as a method of theoretically assessing health behaviours to inform intervention development [21].

The COM-B and TDF analysis was carried out in 4 stages:

1. Examination of the QES data (descriptive and thematic themes) to identify other relevant and/or more specific, factors influencing the target behaviours
2. Identified factors were coded to their relevant COM-B component(s): physical capability, psychological capability, physical opportunity, social opportunity, reflective motivation and automatic motivation
3. Each COM-B component deemed to be relevant (i.e. had at least one factor coded to it) was then taken forward to be explored in more depth through consideration of the TDF domains it is linked to
4. The relevance of each identified TDF domain was then considered in relation to the identified factors.

These four stages identified the relevant psychological domains to be considered for targeting in the intervention.

### Stage 2: identifying intervention content and implementation options

This stage involves using the results of the behavioural diagnosis carried out in Stage 1 to guide decisions regarding the content and delivery of the intervention. This involves first selecting suitable intervention functions (e.g. Education, incentivisation) and policy categories (e.g. guidelines, legislation). (Fig. 1) Based upon these decisions, suitable BCTs are identified for inclusion in the intervention before finally deciding upon a suitable mode of delivery.

#### Steps 4 & 5: identify intervention functions and policy categories

Firstly, the intervention functions (Fig. 1) most suited to target the theoretical domains identified in the COM-B and TDF behavioural analysis carried out in stage 1, were selected using established links between TDF domains and intervention functions [31]. The identified intervention functions suitable in the context of the target behaviours were then considered using APEASE criteria which allowed for evaluation of the appropriateness and suitability of the identified functions for incorporation into the intervention.

Secondly, the seven policy categories (Fig. 1) were then considered using APEASE criteria to identify the categories best suited to deliver the identified intervention functions within the resource constraints of the feasibility trial.

#### Step 6: identify behaviour change techniques

A behaviour change technique taxonomy (BCTTv1) comprised of 93 individual BCTs (e.g. goal-setting), has previously been developed and validated [45]. To guide the identification of all the suitable BCTs from this taxonomy, a table of BCTs appropriate for each intervention function was consulted as suggested in the BCW guidance [42]. The suitability and potential efficacy of each identified BCT was then considered, guided by APEASE, to produce a final set of BCTs for inclusion in the intervention.

#### Step 7: identify the mode of delivery

The final step involves considering the following in relation to intervention delivery: content, provider, recipients, intensity, duration and fidelity. The various potential modes of intervention delivery were considered using the APEASE criteria to assess the options that would be suitable within the constraints and resources of the feasibility trial. The potentially suitable options were then presented to 9 individuals with FH (3 young people and 5 parents) at a patient and public involvement (PPI) which was held as part of the feasibility trial development. Feedback was elicited through open discussion, facilitated by a member of the research team (FK). The feedback obtained was considered by the research team and changes made to the delivery of the intervention.

## Results

### Stage 1: Behavioural diagnosis

#### Step 1: define the problem in behavioural terms & select target behaviour(s)

In general, in the accounts of individuals with FH as captured within the QES synthesis, their dietary or physical activity behaviours are not discussed in the level of detail that the current recommendations are provided. For example, individuals referred to their overall diet, instead of their fat or fibre intake. In recognition of this, these component behaviours were condensed into two broader behavioural targets which were selected as the targets of the intervention: diet and physical activity.

#### Step 2: specify the target behaviours

The target behaviours were specified in detail and the results are displayed in full in Additional file 1 and a brief summary given below.

As FH is chronic condition, the behaviours will need to be performed by individuals or guided by parents every day of their life, starting from the day of their diagnosis which can be at any age from birth. Dietary and physical activity behaviours are performed several times a day, in a variety of different situations, both individually and with others. Consequently, the subsequent behavioural analysis will have to take into consideration the diverse contexts in which individuals will be engaging in these behaviours. A unique consideration for individuals with FH is that they have both unaffected and affected family members – with whom they may be living with and therefore potentially engaging in the behaviours together. This may be especially relevant for children and young adults for whom parents are largely responsible for making dietary choices on their behalf and preparing most meals. Parents also exert influence upon their children’s engagement with physical activities in terms of transportation, financial and scheduling considerations.

#### Step 3: identify what needs to change

Within the data from the QES, in their accounts of managing their FH, individuals infrequently discussed their dietary behaviours in detail and rarely referred to their physical activity behaviours in isolation. Most often, individuals referred to their attempts at living a ‘healthy lifestyle’ which encompasses both behaviours. This made it difficult to differentiate between factors influencing dietary and physical activity behaviours. Therefore, in the reporting of the analysis of the QES findings (displayed in Table 1), factors influencing ‘lifestyle behaviours’ have been reported which refer to individuals engagement with *both* dietary and physical activity treatment guidelines. For instances when it was clear that a factor was related to only the dietary or only the physical activity behaviour, this is specified.

**Table 1:** Full list of factors influencing target behaviour uncovered from the QES

List of specific factors influencing target behaviour^a^	
Young adults struggle to adhere to dietary guidelines when they leave home as they lack the skills to prepare suitable meals themselves	
Individuals learn lifestyle behaviours from their parents	
Lifestyle behaviour habits developed in childhood continue into adulthood	
Inadequate and/or incorrect knowledge of lifestyle guidelines	
Young adults struggle to transition to self-manage condition when they leave home as they lack the knowledge about lifestyle guidelines	
Individuals disregard the role of lifestyle guidelines in the management of their FH, especially when receiving medication	
Individuals find it hard to adhere to the lifestyle guidelines, engaging in behaviours that are not in line with guidelines	
Individuals find it easier to engage in the desired lifestyle behaviours when other family members are engaging in the behaviours too	
Parents concerned about health of their child change their behaviours to facilitate the attainment of lifestyle guidelines by the whole family	
Individuals find it easier to regulate lifestyle behaviours if they have been engaging in them from a young age as they have become habits	
Having practical resources and support to help/guide adherence to lifestyle guidelines	
Individuals faced with other life events (such as illnesses, family bereavement and work/school pressures) do not feel they have the time to focus on engaging in lifestyle behaviours	
Individuals report not being able to adhere to dietary guidelines due to lack of availability of healthy foods and/or high costs of foods	
Individuals find it difficult to adhere to dietary guidelines when in social situations as they don’t want to draw attention to their condition and eat differently from their peers	
Lack of confidence in ability to adhere to lifestyle guidelines as they are perceived to be difficult to follow particularly in certain situations e.g. social occasions, when living independently or when faced with other events in life	
Individuals do not feel FH poses a great risk to their health	
Young people who have not experienced symptoms do not believe that non-adherence with lifestyle guidelines poses risk to their health	
Receiving a formal diagnosis of FH motivates individuals to commence or continue to engage with lifestyle behaviours	
Being actively involved with setting goals for themselves and their children	
Individuals become incentivised to look after their health and engage in lifestyle behaviours when they become responsible for other people e.g. when they become parents	

^a^ Lifestyle guidelines and lifestyle behaviours refer to both dietary and physical activity guidelines or behaviours unless specifically stated to refer to dietary or physical activity guidelines or behaviours

In the subsequent behavioural analysis, all components of the COM-B model were deemed to be relevant and therefore all domains of the TDF were considered in relation to the factors influencing lifestyle behaviours identified from the QES. Twelve of the fourteen TDF domains were found to be relevant for inclusion as targets of the intervention, as detailed in Table 2.

**Table 2:** COM-B and TDF analysis of the identified target behaviours influencing adherence to dietary and physical activity guidelines^a^

COM-B	TDF Domain	Relevance of domain	Appropriate intervention function(s)	Behaviour change technique(s)	Description of how behaviour change technique will be incorporated into intervention
Physical capability	Physical skills	Individuals lack the skills to be able to prepare suitable meals especially for those who are gaining independence during critical periods of transitioning i.e. to live away from home for university	Training	Instruction on how to perform behaviour	Advise individual how to modify current cooking techniques to fit with dietary guidelines i.e. baking instead of frying.
				Demonstration of the behaviour	Individual to be signposted to resources which can aid with demonstration of the behaviour i.e. step by step recipe videos or pictorial guides and parents encouraged to demonstrate cooking skills to CA
				Behavioural practice/rehearsal	Prompt individual to practice cooking during intervention and adults to encourage CA to help them
				Graded tasks	CA to be prompted to help with food preparation -starting with simple task such as preparing vegetables, progressing to more complex tasks until able to prepare entire meal themselves
		Parents impart skills of preparing meals			
					Adults to be prompted to start cooking meals- starting with simple recipes and progressing to more complex ones
				Self-monitoring of behaviour	Individuals asked to record their cooking skills development progress in weekly reflection diaries
Physiological capability	Knowledge	Individuals lack, or have incorrect, knowledge of the guidelines	Education	Instruction on how to perform behaviour	Individual to receive detailed verbal and written information about the lifestyle guidelines and the chance to ask any questions they have- to prevent misinformation being passed to CA. Carried out during initial intervention session and during follow-up sessions.
		Intergenerational transmission of inadequate and/or incorrect knowledge from parent to child			Individuals will receive detailed instructions about food swaps and cooking methods.
					The dietary intake data will be analysed ahead of intervention to establish any existing eating habits that are indicative of misinformation i.e. cooking with coconut oil or eating lots of eggs. This will be proactively brought up for discussion with participant.
	Behavioural regulation	People find it hard to regulate their behaviour because they perceive the lifestyle guidelines to be restrictive and not allow for consumption of foods they enjoy (dietary) and hard to achieve/fit into their lifestyles (physical activity)	Enablement	Self-monitoring of behaviour	Individuals asked to complete weekly reflection diaries in which they record whether they have been able to meet the goals set for lifestyle behaviours which will be discussed during follow-ups
				Goal setting (behavioural)	Individual prompted to set their own goals which they feel are achievable-which take into consideration their food preferences, fitness levels, readiness to change and lifestyles. The goals will be SMART.
				Review behavioural goal(s)	All goals will be reviewed at each follow-up session and, depending on level of achievement, will be increased or stay the same or a new additional goal will be created.
				Action planning	Individual will be prompted to develop specific planning of how they will achieve each goal set i.e. if increasing fibre intake then the food swap or additional food to be included in diet will be specified, along with what meal or snack they will include it in and how many times per day or week.
				Behaviour substitution	Individual and dietitian to brainstorm other foods that they enjoy which are more in line with dietary guidelines which they could choose instead
				Prompts and cues	Individual advised to leave intervention booklet in a place where they regularly eat to prompt them to engage in the agreed dietary goals and set reminders on phones/fitness monitors to move regularly
				Restructuring the physical environment	Advise individual to keep food choices they enjoy eating, that are in line with the guidelines, in the house/at work/in car to encourage consumption of these when they want to eat foods which aren’t necessarily in line with desired behaviours
			Education	Information about antecedents	Individual prompted to consider specific contexts in which they find it hard to adhere to dietary guidelines and identify triggers for this behaviour. The findings will be addressed in action planning and problem-solving session of the intervention.
				Information about emotional consequences	Dietitian to discuss with individual the potential improvement in their mood that they could experience if they choose to engage in dietary behaviours.
					Dietitian to emphasis to individual that if they find foods that they enjoy that fit the guidelines, they will also experience the same enjoyment of these foods as with previous foods
	Cognitive & interpersonal skills	*Not found to be relevant*			
	Memory, attention and decision processes	Individuals who have developed habits in accordance with the physical activity and dietary guidelines find their behaviours easier to regulate since their actions are less focused around conscious decisions and more about habituation	Enablement	Identification of self as a role model	Parents encouraged to view themselves as role models for their children and make their behaviours part of everyday life for their children to help them foster adoption of healthy habits from a young age.
				Behavioural substitution	Individuals encouraged to set goals that involve swapping something they currently do everyday with something that will help them achieve the guidelines to help establish new healthy habits i.e. walking to and from work instead of driving or always having a piece of fruit when they make their morning cup of tea instead of a biscuit.
Social opportunity	Social influences	Easier to follow guidelines if surrounded by family members also doing so	Enablement	Social support (emotional)	Dietitian to provide support during follow-up sessions- individuals advised to keep reflection diaries to record any emotional difficulties they have had which can then be discussed at follow-up.
					Individual also encouraged to seek support from the family member(s) they are taking part in the intervention with- to view it as a ‘team effort’ and provide support and encouragement to each other
		Individuals do not want to draw attention to themselves by eating differently in social situations			
				Social support (practical)	Individuals encouraged to seek support from family members (including those not taking part in intervention) and friends if they are struggling to engage in the behaviours i.e. arranging to go to the gym with a friend or having partner/sibling help with food shopping or meal preparation.
		Children and young adults model what parents do			
					Dietitian will also provide practical support during follow-up sessions- helping individual to identify methods to help encourage the behaviour i.e. links to online resources or suggestions on how they could obtain practical support from friends or family i.e. having their parent prepare help make their lunch
		Individuals find it hard to follow dietary guidelines when with others due to social norms around eating.			
				Identification of self as role model	Individual to communicate to parents how influential their behaviours are to their children and encourage them to engage in the behaviours they want their children to.
		Young adults feel there is stigma around disclosure of their condition and it’s dietary guidelines			Children also encouraged to view themselves as role models for their parents, other siblings and friends. Dietitian to communicate the benefits engaging in the behaviours could bring to these significant others.
				Problem solving	During intervention ‘barriers and solutions’ section, dietitian will encourage individual to think of situations in which they feel they will struggle to engage in the desired behaviours (i.e. social situations) and think of solutions to overcome these. These will be reviewed at follow-up sessions.
		Young adults want to eat with their friends at fast food outlets			
				Restructuring of physical environment	Individuals encouraged to socialise with friends and family in places that facilitate engagement of desired behaviours i.e. meet in the park or choose restaurants in which there are suitable options for them so that they don’t have to draw attention to their food choices.
Physical opportunity	Environmental context and resources	Lack of available healthy food easily accessible within their environment e.g. due to expense or where people typically source food	Training	Instruction on how to perform the behaviour	The instructions provided from dietitian to individual will be individualised in terms of their lifestyles and other conflicting events in life that demand their focus. The advice given will provide instructions to individual about how they can achieve the desired behaviours in their current context i.e. cheaper options for suggested food swaps, suggested food swaps one they can obtain in the current place they shop, easy and quick options for meals if individual has limited time to cook and suggestions of PA they can fit into their current routines such as walking instead of bus/car.
		Conflicting events in life (such as illness or new job) can reduce their available capacity/resources/time to follow the guidelines			
			Enablement	Social support (emotional)	Dietitian to provide emotional support during intervention and follow-up sessions- discussing with them what else is going on in their life and how this is influencing their ability to adhere to guidelines.
					Individuals also encouraged to seek the emotional support of friends and family
				Social support (practical)	The dietitian will encourage the whole family (both with and without FH) to engage in the dietary and PA guidelines to provide support to the individuals taking part in intervention.
		People how have grown up with other family members who have FH have the skills and ability to follow the guidelines themselves			
					Individuals who may struggle to be able to make the changes required to meet the guidelines encouraged to enlist help of family or friends to help them with cooking or food shopping or taking children to sport classes
				Restructuring the social environment	The intervention is delivered at a family-based level with parent and child making dietary and PA choices together. Any other family members will also be encouraged to make the changes aswell to facilitate a home environment that encourages adherence to the dietary and PA guidelines.
Reflective motivation	Professional/social role and identity	When they had a genetic diagnosis, as opposed to a clinical diagnosis of ‘possible’ FH, individuals feel that following guidelines are a natural part of their identity as someone with FH	Persuasion	Framing/reframing	Guidelines communicated to individual as being specifically for individuals with FH as opposed to general healthy lifestyle guidelines provided to all individuals. The inclusion of two specific dietary guidelines for individuals with FH such as eating foods fortified with plant stanols/sterols and reducing dietary cholesterol intakes will help individuals to buy into the idea that following the guidelines is part of their identity of having FH. The benefits of following the guidelines in the management of their FH will be emphasised, in addition to general overall health benefits.
	Beliefs about capabilities	Lack of confidence in ability to adhere to guidelines as they are perceived as being restrictive (dietary) and difficult to follow (dietary and PA)	Persuasion	Framing/reframing	Dietary guidelines to be communicated as a healthy lifestyle rather than a restrictive diet, with all foods permitted. Emphasis will be put on foods to add into the diet (fruits, vegetables, fibre rich foods, plant sterols/stanols) to help individual view dietary choices are positive and enjoyable.
					Physical activity to be communicated positively, with emphasis placed upon finding activities that the individual enjoys doing, either alone or with friends or family, rather than it being a chore they have to try and fit into their day without enjoying it.
				Verbal persuasion about capabilities	Dietitian to encourage individual and tell them that they are capable of changing their behaviours- to be included during initial session and follow-ups.
				Focus on past success	Individuals asked to keep weekly reflection diaries to record progress in between follow-ups. Dietitian to ask during follow-up what the individual has recorded and to focus on any successful changes they have made and refer back to these to provide encouragement and motivation to individual to carry on and make more changes.
				Feedback on outcomes of behaviour	Individual to receive feedback about how their dietary intakes and PA levels compare to the guidelines at the start and end of the intervention as evidence of their capability of adhering to the guidelines to promote maintenance of behaviours after intervention ended.
	Beliefs about consequences	Not believing that FH poses a health risk so they do not feel the need to follow guidelines.	Education	Information about health consequences	The intervention starts with explanation from dietitian about the importance of lifestyle guidelines in the management of FH. It will be explained that despite use of medication, many individuals with FH may still be at higher risk of cardiovascular disease and adherence to lifestyle guidelines can help reduce this risk. The benefits to their overall health will also be communicated. Individuals to be informed about the ‘silent’ nature of cholesterol and the importance of keeping cholesterol as low as possible for your whole life, before any symptoms occur.
		Especially salient in people who are asymptomatic and people who do not have a family history of FH			
			Persuasion	Credible sources	Intervention delivered by a dietitian who will explain the training they have undertaken to gain that title- to help individual recognise that their advice is credible. All individuals will also be informed that their doctor is aware and supportive of them receiving the intervention as they view it as being part of their clinical care.
		As they are taking medication, they feel absolved from the need for following the guidelines.			
					Individual to be receive verbal and written advice about the guidelines which will include information about where the guidelines have come from- national committees across several countries who have reviewed all the available scientific evidence and come to same conclusion about the guidelines that individuals with FH should be following.
		Individuals do not believe that following the guidelines will impact on their health outcomes.			
				Biofeedback	Individual to be provided with weight, body fat % and blood pressure before starting the intervention to prompt adoption of guidelines- either to improve these figures or maintain them. Also will be provided with this information at the end of the intervention to compare with their results at the start of the intervention which will be discussed in relation to the behaviour changes made over the intervention.
	Intentions	When people become parents, their intentions to look after their own health increases as they feel responsible to live longer for their children	Persuasion	Comparative imaging of future outcomes	Dietitian to prompt individual to think about what the possible health outcomes would be if they choose to follow guidelines vs if they chose not to- with emphasis on what this would mean for their children or parents.
				Identification of self as role model	Dietitian to emphasis to parent that CA are heavily influenced by their behaviours, to encourage them to engage in guidelines for the sake of their childs health.
				Information about health consequences	Individuals to be informed about the ‘silent’ nature of cholesterol and the importance of keeping cholesterol as low as possible for your whole life, before any symptoms occur. The importance of the role of lifestyle guidelines to be discussed in context of benefits they provide over and above medication alone.
		Children and young adults do not have the intention to deal with their condition yet			
	Goals	Individuals desire to be actively involved in treatment decisions for themselves and their children	Enablement	Goal setting (behavioural)	Individuals will decide (with help from dietitian and family members) upon goals for each of the guidelines and will be recorded in their intervention booklets. These goals will be SMART in nature and will be tailored toward the individuals individual circumstances, lifestyles and food preferences and readiness to change. Goals will not be prescribed- individual encouraged to think of them themselves.
					Parents will be encouraged to help CA with setting their goals.
				Review goal (behavioural)	At each follow-up session, dietitian and individual will review the goals set at previous session or follow-up. Together they will agree to either: keep goal the same, modify the goal or create new goal. These decisions will be based upon individuals levels of achievement and willingness to change.
				Self-monitoring of behaviour	Individual asked to record their achievement with the goals they set in weekly reflection diaries to be discussed at each follow-up sessions.
	Optimism	*Not found to be relevant*			
Automatic motivation	Reinforcement	Parents are incentivised to enable child to follow guidelines to ensure that the child will achieve the best possible health outcomes	Persuasion	Identification of self as role model	Dietitian to emphasis to parent that CA are heavily influenced by their behaviours, to encourage them to engage in lifestyle guidelines for the sake of their childs health.
				Comparative imaging of future outcomes	Dietitian to prompt individual to think about what the possible health outcomes would be if they choose to follow lifestyle guidelines vs if they chose not to- with emphasis on what this would mean for their children or parents.
		Parents are also incentivised to take care of themselves so that they are healthy and able to take care of their child.		Framing/ reframing	Individual prompted to view engaging in the behaviours as something that will benefit their children, rather than just themselves.
			Enablement	Social support (emotional)	Dietitian to encourage parent and CA to provide support to each other throughout the intervention and encourage each other to engage in the behaviours
	Emotion	Individuals who themselves, or their family members, have experienced CVD symptoms experience stress and anxiety about the long-term health outcomes related to FH	Persuasion	Framing/ reframing	Individual prompted to view the guidelines as behaviours that can help reduce their risk of developing symptoms as their family members have, or reduce the likelihood of experiencing further symptoms. They are something the individual can do to help take control of their health.
				Comparative imaging of future outcomes	Dietitian to prompt individual to think about what the possible health outcomes would be if they choose to follow guidelines vs if they chose not to.
				Social support (emotional)	Dietitian to encourage parent and CA to provide support to each other throughout the intervention and encourage each other to engage in the guidelines

^a^ ‘guidelines’ refers to both dietary and physical activity guidelines. In instances where only dietary or only physical activity guidelines are being referred to, this is specifically stated. CA: children and adolescents; SMART: specific, measurable, acceptable, realistic, time based

### Stage 2: identifying suitable intervention content and implementation options

#### Steps 4 & 5: identify intervention functions and policy categories

Seven of the nine intervention functions were identified as being appropriate to target diet and physical activity behaviour change in the current population and after consideration of the APEASE criteria, four were deemed to be suitable for inclusion: Education, training, enablement and persuasion. Additional file 2 displays the details of the APEASE evaluation carried out on the seven candidate intervention functions.

Guided by the policy categories suggested by the BCW to deliver each of the four identified intervention functions, all seven category options were found to be suitable. After consideration of APEASE criteria, guidelines and service provision categories were identified as being suitable for delivering the intervention functions in the context of the feasibility trial (ISRCTN24880714). ‘Service provision’ will be in the format of setting up a dietetic service to deliver the diet and physical activity intervention and ‘guidelines’ will be in the format of a suggested protocol for the delivery and content of the intervention, to be implemented in lipid clinics, subject to the outcomes of the feasibility trial.

#### Step 6: identify behaviour change techniques

Table 2 displays the twenty-six BCTs identified for inclusion in the intervention following evaluation using the APEASE criteria. Each BCT aims to addresses *both* physical activity and dietary behaviours unless specifically stated otherwise. Details of how each BCT will be incorporated into the intervention are displayed in Table 2.

#### Step 7: identify the mode of delivery

The decisions regarding mode of delivery have been broken down into: *Provider, Recipients, Intensity* and *Fidelity.*

##### Provider

The current recommendations for provision of care to individuals with FH in England state that the lifestyle advice should be delivered by a healthcare professional with the relevant knowledge in nutrition [13] and therefore it was decided that a dietitian would deliver the intervention.

##### Recipients

The greatest reductions in CVD risk in individuals with FH are evident in those who commence treatment from a young age [46, 47] and thus, the research team decided the intervention would have most benefit if delivered to children and adolescents with FH. The genetic pattern of inheritance means that every individual with FH will have one affected parent, and findings from the QES highlight the importance of establishing healthy lifestyle behaviour habits from a young age and having other family members engaging in the behaviours. These findings suggest that delivering the intervention to families may increase the chances of behaviour change and therefore the intervention is being delivered to parent and child dyads.

##### Intensity

With an estimated prevalence of 1 in 250 [5], it is essential that the intervention is pragmatic with the potential to be delivered within the time and budget constraints of current health services. As an initial dietetic consultation usually takes 30 min, the decision was made for the intervention to be delivered in 60 min to optimise delivery to two individuals. The PPI group feedback was that a one-hour session would be acceptable, but that they may struggle with motivation and suggested follow-up sessions. Participants stated that it may be difficult to attend more visits to the hospital and suggested that they would prefer telephone follow-ups. This was considered more appropriate than emails as many said they did not regularly check their personal emails. Based on these findings, the intervention is through a one-hour face to face consultation and four follow-up phone calls. For the purpose of evaluation within the feasibility trial, the follow-up period is 12 weeks.

##### Fidelity

To ensure the intervention is delivered as intended, checklists were created for completion by the dietitian during each initial and follow-up session (Additional file 3). These checklists provide detailed information about the content that is intended to be covered during each session. A brief overview of the intended intervention content and the aims for the participant are displayed in Table 3, alongside the BCTs intended to be incorporated for each section of the initial session and the follow up sessions. The level of detail in the checklist provided to the dietitian is intended to act as a script for the sessions with suggested prompts and questions provided at relevant points to facilitate participant involvement. To facilitate the subsequent evaluation of the intervention, alongside completion of the checklists, the dietitian completes a detailed reflection of each session in which they will record details of content covered and the BCTs utilised by themselves and the participants. To further facilitate the intended delivery of the intervention content and enable individuals to engage in the lifestyle behaviours on their own at home, age appropriate intervention booklets were designed: one for children 10–13 years and one for individuals aged 14 years and over. (Additional file 4).

**Table 3:** Detailed break-down of intervention content and incorporated BCTs

Section	Aim(s) for participants	Incorporated BCTs
Scientific rationale	To understand the importance of diet and physical activity in the management of FH and for their overall health.	Information about health consequences, credible sources, biofeedback, information about emotional consequences, identification of self as a role model, comparative imaging of future outcomes
	To be aware of the importance placed on diet and PA by national and international guidelines for FH and the current recommendations that all FH patients should receive individualised advice about diet & physical activity	
	To understand that the earlier treatment for FH starts, the more effective it is, and this is why it is important to optimise diet and physical activity from a young age	
Dietary guidelines education	To increase knowledge of what a healthy balanced diet looks like, including the food groups and the proportion each one should make to diet	Instruction on how to perform behaviour, demonstration of the behaviour, behavioural practice/rehearsal, behaviour substitution, framing/re-framing
	To increase knowledge of what the 5 dietary targets of the intervention are	
	To understand why each target is important for their health and for the management of their FH	
	To understand what foods to include/exclude and/or increase/decrease consumption of to achieve targets	
Physical activity guidelines education	To increase knowledge of what the physical activity recommendations are for the individuals age	
	To increase knowledge of physical activity intensities (i.e. low, moderate and vigorous) and what types of activity fall into these intensity groups	
	To understand how to incorporate more physical activity into everyday life to help increase levels to recommended amounts (or more)	
Goal setting	Work with the dietitian to develop SMART goals for each target behaviour. These will be changes to their lifestyle that they agree to make over the following 12 weeks to achieve nutritional intakes and PA levels closer to the targets.	Goal setting (behavioural), behaviour substitution, action planning, prompts and cues, Restructuring the physical environment, identification of self as a role model, Restructuring the social environment
Barriers and solutions	To identify potential barriers that may prevent individuals from meeting their goals	behaviour substitution, behaviour substitution, prompts and cues, Information about antecedents, Restructuring the physical environment, problem solving, Restructuring the social environment
	To identify, through discussion with dietitian and family, solutions	
Wrap up & instructions	An opportunity to ask any unanswered questions	Social support (emotional), verbal persuasion about capabilities
	Receive encouragement & motivation from dietitian	
	To understand the purpose of the weekly reflection diaries and know how to fill them out	
Follow-up sessions (weeks 2,4, 8 and 11)	To review with dietitian their progress towards goals set at previous session	Self-monitoring of behaviour, review behavioural goal(s), behaviour substitution, behaviour substitution, prompts and cues, Information about antecedents, Restructuring the physical environment, identification of self as a role model, problem solving, social support (practical), Social support (emotional), verbal persuasion about capabilities, Focus on past success, Feedback on outcomes of behaviour
	To adjust goals accordingly, with help of dietitian, to facilitate attainment	
	Brainstorm solutions to any identified barriers to achievement recorded in reflection diaries	
	To receive encouragement and motivation from dietitian	

*SMART* specific, measurable, acceptable, realistic, time based

## Discussion

This paper reports the systematic development of an intervention designed to improve the dietary and physical activity behaviours in individuals with FH. A behavioural analysis was carried out upon the findings of a QES following the steps outlined in the Behaviour Change Wheel (BCW) guidance. In addition, a taxonomy of behaviour change (BCTTv1) was applied to select the Behaviour Change Techniques (BCTs) most appropriate to bring about the desired changes in behaviour and inform the content of the intervention. To our knowledge this is the first study to use this approach in the development of a behaviour change intervention for individuals with FH. It is anticipated that the use of this systematic approach will increase the potential efficacy of the intervention and enable an evaluation to be conducted to gain a comprehensive understanding of the intervention effects.

Twelve of the fourteen domains from the Theoretical Domains Framework (TDF) were found to be relevant to dietary and physical activity behaviours in individuals with FH. These behaviours have been reported to be influenced by several of the same theoretical constructs in research carried out in individuals with other chronic conditions including Type II diabetes [48] and kidney disease [49] and in those at risk of cardiovascular disease [50]. In all cases, multiple theoretical constructs were found to be relevant, demonstrating the multifactorial nature of these behaviours and the importance of considering a wide range of potential influences when developing interventions. The present research identified additional relevant constructs such as *beliefs about consequences* not found in Type II diabetes and *reinforcement* and *emotion* not identified in those with chronic kidney disease. This may reflect the unique context that living with FH presents and/or our analysis of QES findings which represents the experiences of a wider range of individuals.

Of the research discussed, the additional step whereby identified theoretical constructs are linked to active ingredients for use in an intervention has only reported for those at high risk of CVD. This research identified 12 BCTs for inclusion, of which 10 were BCTs included in the current intervention. Systematic reviews of interventions targeting physical activity and dietary behaviours which retrospectively identified BCTs have reported a wide range of BCTs across interventions, the majority of which were identified in the current research [25, 28, 29, 51, 52]. In all cases, the additional BCTs used in previous, or suggested for use in future interventions, were identified as being relevant but not included in the current research due to not meeting APEASE criteria. This included *biofeedback* and techniques related to *incentivisation* and *restriction* intervention functions. All five systematic reviews concluded that previous interventions have not been adequately described to allow for reliable analysis of efficacy and that the design and content of all future interventions should be explicitly reported [25, 28, 29, 51, 52]. The systematic review conducted by the American Heart Association (AHA) provided graded evidence-based recommendations from their findings and concluded there was most evidence to support the inclusion of BCTs related to goal setting, feedback on goals, self-monitoring and/or self-efficacy [52]. While some BCTs were more frequently included, such as those related to goals, monitoring, knowledge and feedback, there was insufficient evidence from the other four systematic reviews to identify which BCTs would be most effective. Furthermore, all reviews included only trials involving adults and therefore their results may not be transferable for application in this trial which aims to target the behaviours of children and adults. This is the rationale behind the decision to include all twenty-six BCTs that met the APEASE criteria in the current intervention.

A previous intervention designed to improve the dietary and physical activity behaviours of individuals with FH shared many similarities to the current intervention [22]. Participants received an hour-long individualised counselling session followed by telephone follow-ups in addition to access to online learning modules. Many of the theoretical constructs that were targeted were similar to the current intervention as were the techniques to bring about change such as *action planning, goal setting and information about health consequences* [22]. However, the findings from the process evaluation suggest the intervention was not successful in increasing motivation to change, with less than a third of participants completing any of the available online modules and making action plans [53]. This may be explained by the assumption of the *I-Change* model used in intervention development that increasing knowledge and awareness alone are capable of evoking sufficient motivation to promote engagement in the behaviours. The behavioural analysis undertaken in the present study found the reflective motivation component and its component domains are important theoretical constructs to target in individuals with FH. It may be that these constructs were not adequately considered in the design of the previous intervention [22]. With the inclusion of several BCTs to target these constructs in the present intervention it is anticipated that individuals will be motivated to engage and proceed to developing their capability to achieve the desired changes in behaviour.

### Strengths

The QES synthesised the experiences and beliefs of 264 individuals with FH, and 13 of their family members, from eight countries. This provided a rich insight into the unique context in which individuals with FH engage in the behaviours targeted by the intervention. It is anticipated that this approach will increase the efficacy of the intervention to bring about behaviour change in a wider range of individuals, as opposed to an intervention based upon the findings of a single qualitative study. The behavioural analysis conducted provided a systematic method of analysis to gain a theoretical understanding of the behaviours and the factors influencing them. This allowed for identification of the relevant theoretical constructs to target, and the most appropriate techniques to do so, in order to evoke behaviour change in this specific population group. This overcomes the limitations of applying a fixed theoretical model of behaviour change which may not address the unique situations faced by individuals with FH.

### Limitations

Firstly, although findings from the QES, on which the intervention was based, overcame some of the transferability issues of individual qualitative studies [38] the sample represents only individuals from developed countries, most of whom were highly educated and white European. Therefore, the active components of the intervention may not address all the theoretical constructs required to bring about behaviour change in individuals from different backgrounds.

Secondly, the BCTs for each identified intervention function were selected using links proposed and suggested for use in the original BCW guidance [42]. This was conducted prior to becoming aware of the mapping work which is currently being undertaken to further understand and define the links between BCTs and theoretical constructs [54–56]. Any future research should use the newly available Theory and Techniques tool (https://theoryandtechniquetool.humanbehaviourchange.org/) when selecting and evaluating the BCTs included in an intervention.

### Further research

The developed intervention will now undergo a comprehensive evaluation to determine its potential efficacy and acceptability within a randomised controlled pilot feasibility trial (ISRCTN24880714). In recognition of the limitations described above, the proposed feasibility trial aims to recruit individuals from a wide variety of social and ethnic backgrounds to determine if these factors affect recruitment and intervention efficacy. The objective measures of physical activity and the online dietary intake tool which are being utilised in the feasibility trial are validated methods and will provide reliable measures of the efficacy of the intervention.

There is currently no gold standard approach to assessing the effectiveness of specific, or combinations of, BCTs in research trials [57]. Given the large amount of BCTs included, the initial evaluation carried out upon the results of the pilot trial will focus upon the feasibility and acceptability of delivering the intervention as proposed. These will be assessed using the fidelity checklists and detailed reflections completed by the dietitians following each intervention session and qualitative interviews conducted with a sub-sample of the participants. These data will be examined to identify the frequency with which each BCT was included in the delivery of the intervention by the dietitians and the usage frequency of the BCTs requiring participant engagement by the participants throughout the intervention period. The data will also be examined to identify the BCTs that were perceived to be fundamental in promoting behaviour change by both the dietitians and the participants. These results will then also be used to conduct preliminary analysis on the potential efficacy of individual, or groups of BCTs, selected. This will be carried out through exploration of the effect of the omission of any BCT(s) by the dietitian (as captured by fidelity checklist and reflections) or self-reported non engagement with any BCT(s) (as captured through qualitative interviews and dietitian reflections). The inter-individual variation in participant engagement or BCT effectiveness will be explored to understand the effect of factors such as age and gender. These results will enable refinement of the intervention, if required, before undergoing a more comprehensive evaluation in a fully powered randomised controlled trial.

## Conclusion

The novel application of the Behaviour Change Wheel to the findings of a qualitative evidence synthesis has enabled the development of a behaviour change intervention targeting dietary and physical activity behaviours in children and adults with FH. The explicit account of the development process and content of this intervention faciltates the application of a subsequent in-depth evaluation to determine the efficacy of the BCTs selected for inclusion. This evaulation is now being carried out as part of a feasibility trial which will allow this intervention to be refined and replicated in other contexts. Furthermore, this clear and transparent report of the development of an intervention for individuals with FH can be used to guide intervention development by researchers in other healthcare contexts.

## Supplementary information

**Additional file 1.** Behaviour specifications of the target behaviours.

**Additional file 2.** APEASE criteria evaluation of candidate intervention functions.

**Additional file 3.** Intervention checklist for 1st session

**Additional file 4.** Intervention booklet for participants aged 14 years and above

## Data Availability

The dataset(s) supporting the conclusions of this article are included within the article and its additional file(s).

## Abbreviations

AHA: American Heart Association
APEASE: Affordability, practicality, effectiveness/cost-effectiveness, acceptability, side effects, equity
BCT: Behaviour Change Technique
BCTtV1: Behaviour Change Technique Taxonomy Version 1
BCW: Behaviour Change Wheel
CA: Children and adolescents
COM-B: Capability, opportunity, motivation-behaviour
CVD: Cardiovascular disease
FH: Familial hypercholesteroleamia
HCP: Healthcare professional
LDL-C: Low density lipoprotein cholesterol
MRC: Medical Research Council
NHS: National Health Service
QES: Qualitative evidence synthesis
RCT: Randomised controlled trial
SMART: Specific, measurable, acceptable, realistic, time based.
TDF: Theoretical Domains Framework
TEI: Total energy intake

## References

[1] Goldstein JL, Hobbs HH, Brown MS, Scriver CR, Beaudet AL, Sly WS, Valle D (2001). Familial hypercholesterolemia. The metabolic and molecular bases of inherited disease.

[2] Wong B, Kruse G, Kutikova L, Ray KK, Mata P, Bruckert E (2016). Cardiovascular disease risk associated with familial hypercholesterolemia: a systematic review of the literature. Clin Ther.

[3] Retterstol K, Mundal L, Igland J, Seppola Tell G, Holven K, Braglien Veierød M (2017). Incidence of various types of atherosclerotic disease in patients with genotyped familial hypercholesterolemia. Atherosclerosis.

[4] Galema-Boers AM, Lenzen MJ, Engelkes SR, Sijbrands EJ (2018). Roeters van Lennep JE. Cardiovascular risk in patients with familial hypercholesterolemia using optimal lipid-lowering therapy. J Clin Lipidol.

[5] Akioyamen Leo E, Genest Jacques, Shan Shubham D, Reel Rachel L, Albaum Jordan M, Chu Anna, Tu Jack V (2017). Estimating the prevalence of heterozygous familial hypercholesterolaemia: a systematic review and meta-analysis. BMJ Open.

[6] Vallejo-Vaz AJ, De Marco M, Stevens CAT, Akram A, Freiberger T, Hovingh GK (2018). Overview of the current status of familial hypercholesterolaemia care in over 60 countries - the EAS familial Hypercholesterolaemia studies collaboration (FHSC). Atherosclerosis..

[7] Iyen B, Qureshi N, Kai J, Akyea RK, Leonardi-Bee J, Roderick P (2019). Risk of cardiovascular disease outcomes in primary care subjects with familial hypercholesterolaemia: a cohort study. Atherosclerosis.

[8] Pérez de Isla L, Alonso R, Mata N, Fernández-Pérez C, Muñiz O, Díaz-Díaz JL (2017). Predicting Cardiovascular Events in Familial Hypercholesterolemia. Circulation.

[9] Akioyamen Leo E., Genest Jacques, Chu Anna, Inibhunu Happy, Ko Dennis T., Tu Jack V. (2019). Risk factors for cardiovascular disease in heterozygous familial hypercholesterolemia: A systematic review and meta-analysis. Journal of Clinical Lipidology.

[10] Arnett Donna K, Blumenthal Roger S, Albert Michelle A, Buroker Andrew B, Goldberger Zachary D, Hahn Ellen J (2019). 2019 ACC/AHA guideline on the primary prevention of cardiovascular disease. Circulation.

[11] Piepoli MF, Hoes AW, Agewall S, Albus C, Brotons C, Catapano AL (2016). 2016 European Guidelines on cardiovascular disease prevention in clinical practice: The Sixth Joint Task Force of the European Society of Cardiology and Other Societies on Cardiovascular Disease Prevention in Clinical Practice (constituted by representatives of 10 societies and by invited experts) Developed with the special contribution of the European Association for Cardiovascular Prevention & Rehabilitation (EACPR). Eur Heart J.

[12] Catapano AL, Graham I, De Backer G, Wiklund O, Chapman MJ, Drexel H (2016). 2016 ESC/EAS guidelines for the Management of Dyslipidaemias. Eur Heart J.

[13] NICE clinical guideline 71: Familial hypercholesterolemia: identification and management. [Internet]. 2008 [cited 19/01/2018]. Available from: http://nice.org.uk/guidance/cg71.

[14] Claassen L, Henneman L, Kindt I, Marteau TM, Timmermans DRM (2010). Perceived risk and representations of cardiovascular disease and preventive behaviour in people diagnosed with familial hypercholesterolemia: a cross-sectional questionnaire study. J Health Psychol.

[15] Avis HJ, Kusters DM, Vissers MN, Huijgen R, Janssen TH, Wiegman A (2012). Follow-up of children diagnosed with familial hypercholesterolemia in a National Genetic Screening Program. J Pediatr.

[16] Kinnear FJ, Wainwright E, Perry R, Lithander FE, Bayly G, Huntley A (2019). Enablers and barriers to treatment adherence in heterozygous familial hypercholesterolaemia: a qualitative evidence synthesis. BMJ Open.

[17] Craig Peter, Dieppe Paul, Macintyre Sally, Michie Susan, Nazareth Irwin, Petticrew Mark et al. Developing and evaluating complex interventions: the new Medical Research Council guidance. BMJ. 2008;337:a1655. 10.1136/bmj.a1655 PMC2769032 18824488

[18] Cathain A, Croot L, Duncan E, Rousseau N, Sworn K, Turner KM (2019). Guidance on how to develop complex interventions to improve health and healthcare. BMJ Open.

[19] Moore G, Audrey S, Barker M, Bond L, Bonell C, Cooper C (2014). Process evaluation in complex public health intervention studies: the need for guidance. J Epidemiol Community Health.

[20] Moore GF, Audrey S, Barker M, Bond L, Bonell C, Hardeman W (2015). Process evaluation of complex interventions: Medical Research Council guidance. BMJ.

[21] Cane J, O’Connor D, Michie S (2012). Validation of the theoretical domains framework for use in behaviour change and implementation research. Implement Sci.

[22] Broekhuizen K, van Poppel MN, Koppes LL, Brug J, van Mechelen W (2010). A tailored lifestyle intervention to reduce the cardiovascular disease risk of individuals with familial hypercholesterolemia (FH): design of the PRO-FIT randomised controlled trial. BMC Public Health.

[23] Broekhuizen K, van Poppel MN, Koppes LL, Kindt I, Brug J, van Mechelen W (2012). Can multiple lifestyle behaviours be improved in people with familial hypercholesterolemia? Results of a parallel randomised controlled trial. PLoS One.

[24] de Vries H, Mesters I, Jv R, Willems K, Reubsaet A (2006). Motives of Belgian Adolescents for Using Sunscreen: The Role of Action Plans. Cancer Epidemiol Biomark Prev.

[25] Desroches S, Lapointe A, Ratté S, Gravel K, Légaré F, Turcotte S. Interventions to enhance adherence to dietary advice for preventing and managing chronic diseases in adults. Cochrane Database Syst Rev. 2013;(2):CD008722. 10.1002/14651858.CD008722.pub2.. PMID: 23450587; PMCID: PMC4900876. 10.1002/14651858.CD008722.pub2 PMC4900876 23450587

[26] Conn VS, Hafdahl AR, Brown SA, Brown LM (2008). Meta-analysis of patient education interventions to increase physical activity among chronically ill adults. Patient Educ Couns.

[27] Prestwich A, Sniehotta FF, Whittington C, Dombrowski SU, Rogers L, Michie S (2014). Does theory influence the effectiveness of health behavior interventions? Meta-analysis. Health Psychol.

[28] Greaves CJ, Sheppard KE, Abraham C, Hardeman W, Roden M, Evans PH (2011). Systematic review of reviews of intervention components associated with increased effectiveness in dietary and physical activity interventions. BMC Public Health.

[29] Patnode CD, Evans CV, Senger CA, Redmond N, Lin JS (2017). U.S. Preventive Services Task Force Evidence Syntheses, formerly Systematic Evidence Reviews. Behavioral Counseling to Promote a Healthful Diet and Physical Activity for Cardiovascular Disease Prevention in Adults Without Known Cardiovascular Disease Risk Factors. Updated Systematic Review for the US Preventive Services Task Force.

[30] Cane J, Richardson M, Johnston M, Ladha R, Michie S (2015). From lists of behaviour change techniques (BCTs) to structured hierarchies: comparison of two methods of developing a hierarchy of BCTs. Br J Health Psychol.

[31] Michie S, van Stralen MM, West R (2011). The behaviour change wheel: a new method for characterising and designing behaviour change interventions. Implement Sci.

[32] Glasziou P, Meats E, Heneghan C, Shepperd S (2008). What is missing from descriptions of treatment in trials and reviews?. BMJ.

[33] Michie S, Abraham C (2004). Interventions to change health behaviours: evidence-based or evidence-inspired?. Psychol Health.

[34] Craig LE, Taylor N, Grimley R, Cadilhac DA, McInnes E, Phillips R (2017). Development of a theory-informed implementation intervention to improve the triage, treatment and transfer of stroke patients in emergency departments using the theoretical domains framework (TDF): the T(3) trial. Implement Sci.

[35] Steinmo S, Fuller C, Stone SP, Michie S (2015). Characterising an implementation intervention in terms of behaviour change techniques and theory: the ‘Sepsis six’ clinical care bundle. Implement Sci.

[36] Munir F, Biddle SJH, Davies MJ, Dunstan D, Esliger D, Gray LJ (2018). Stand more AT work (SMArT work): using the behaviour change wheel to develop an intervention to reduce sitting time in the workplace. BMC Public Health.

[37] Flemming K, Booth A, Garside R, Tunçalp Ö, Noyes J (2019). Qualitative evidence synthesis for complex interventions and guideline development: clarification of the purpose, designs and relevant methods. BMJ Glob Health.

[38] Finfgeld-Connett D (2010). Generalizability and transferability of meta-synthesis research findings. J Adv Nurs.

[39] Lozano P, Henrikson NB, Dunn J, Morrison CC, Nguyen M, Blasi PR (2016). U.S. Preventive Services Task Force Evidence Syntheses, formerly Systematic Evidence Reviews. Lipid Screening in Childhood and Adolescence for Detection of Familial Hypercholesterolemia. A Systematic Evidence Review for the US Preventive Services Task Force.

[40] Malhotra A, Shafiq N, Arora A, Singh M, Kumar R, Malhotra S (2014). Dietary interventions (plant sterols, stanols, omega-3 fatty acids, soy protein and dietary fibers) for familial hypercholesterolaemia. Cochrane Database Syst Rev.

[41] Broekhuizen K, van Poppel MN, Koppes LL, Kindt I, Brug J, van Mechelen W (2012). No significant improvement of cardiovascular disease risk indicators by a lifestyle intervention in people with familial hypercholesterolemia compared to usual care: results of a randomised controlled trial. BMC Res Notes.

[42] Michie S, Atkins L, West R. The Behaviour Change Wheel. A guide to Designing Interventions. London: Silverback Publishing 2014; 2014.

[43] Kinnear FJ, Perry R, Searle A, Hamilton-Shield JP, Lithander FE (2018). How do the experiences and beliefs of adults and children with heterozygous familial hypercholesterolaemia influence their adherence to treatment? A systematic review of qualitative evidence protocol. Syst Rev.

[44] 2019 ESC/EAS guidelines for the management of dyslipidaemias: Lipid modification to reduce cardiovascular risk. Atherosclerosis. 2019;290:140–205. 10.1016/j.atherosclerosis.2019.08.014 31591002

[45] Michie S, Richardson M, Johnston M, Abraham C, Francis J, Hardeman W (2013). The behavior change technique taxonomy (v1) of 93 hierarchically clustered techniques: building an international consensus for the reporting of behavior change interventions. Ann Behav Med.

[46] Perez-Calahorra Sofía, Laclaustra Martín, Marco-Benedí Victoria, Lamiquiz-Moneo Itziar, Pedro-Botet Juan, Plana Núria, Sanchez-Hernandez Rosa M., Amor Antonio J., Almagro Fátima, Fuentes Francisco, Suarez-Tembra Manuel, Civeira Fernando (2019). Effect of lipid-lowering treatment in cardiovascular disease prevalence in familial hypercholesterolemia. Atherosclerosis.

[47] Masana Luís, Zamora Alberto, Plana Núria, Comas-Cufí Marc, Garcia-Gil Maria, Martí-Lluch Ruth, Ponjoan Anna, Alves-Cabratosa Lia, Elosua Roberto, Marrugat Jaume, Dégano Irene R., Ramos Rafel (2019). Incidence of Cardiovascular Disease in Patients with Familial Hypercholesterolemia Phenotype: Analysis of 5 Years Follow-Up of Real-World Data from More than 1.5 Million Patients. Journal of Clinical Medicine.

[48] Penn L, Dombrowski SU, Sniehotta FF, White M (2013). Participants’ perspectives on making and maintaining behavioural changes in a lifestyle intervention for type 2 diabetes prevention: a qualitative study using the theory domain framework. BMJ Open.

[49] Baay S, Hemmelgarn B, Tam-Tham H, Finlay J, Elliott MJ, Straus S (2019). Understanding adults with chronic kidney disease and their caregivers’ self-management experiences: a qualitative study using the theoretical domains framework. Can J Kidney Health Dis.

[50] McEvoy CT, Moore SE, Appleton KM, Cupples ME, Erwin C, Kee F (2018). Development of a peer support intervention to encourage dietary behaviour change towards a Mediterranean diet in adults at high cardiovascular risk. BMC Public Health.

[51] Fappa E, Yannakoulia M, Pitsavos C, Skoumas I, Valourdou S, Stefanadis C (2008). Lifestyle intervention in the management of metabolic syndrome: could we improve adherence issues?. Nutrition (Burbank, Los Angeles County, Calif).

[52] Artinian NT, Fletcher GF, Mozaffarian D, Kris-Etherton P, Van Horn L, Lichtenstein AH (2010). Interventions to promote physical activity and dietary lifestyle changes for cardiovascular risk factor reduction in adults: a scientific statement from the American Heart Association. Circulation.

[53] Broekhuizen K, Jelsma Gm J, van PoppelNm M, Koppes Lj L, Brug J, van Mechelen W (2012). Is the process of delivery of an individually tailored lifestyle intervention associated with improvements in LDL cholesterol and multiple lifestyle behaviours in people with familial hypercholesterolemia?. BMC Public Health.

[54] Carey RN, Connell LE, Johnston M, Rothman AJ, de Bruin M, Kelly MP (2018). Behavior change techniques and their mechanisms of action: a synthesis of links described in published intervention literature. Ann Behav Med.

[55] Connell Bohlen L, Carey RN, de Bruin M, Rothman A, Johnston M, Kelly MP, Michie S. Links between behaviour change techniques and mechanisms of action: an expert consensus study. PsyArXiv Epub ahead of print. 2018. 10.1093/abm/kay082 PMC6636885 30452535

[56] Johnston M, Rachel N, Carey LCB, Johnston DW, Rothman A, de Bruin M, Kelly MP, et al. Linking Behavior Change Techniques and Mechanisms of Action: Triangulation of Findings from Literature Synthesis and Expert Consensus. PsyArXiv. 2018; Epub ahead of print.

[57] Michie S, West R, Sheals K, Godinho CA (2018). Evaluating the effectiveness of behavior change techniques in health-related behavior: a scoping review of methods used. Transl Behav Med.

