# Supplementary material for: The development of a theory informed behaviour change intervention to improve adherence to dietary and physical activity treatment guidelines in individuals with familial hypercholesterolaemia (FH)
Source: BMC Health Serv Res. 2020 Jan 8;20:27. doi: 10.1186/s12913-019-4869-4 (PMC6950899; doi:10.1186/s12913-019-4869-4)
Supplement: Supplementary file 1 — Additional file 1. Behaviour specifications of the target behaviours. [file 12913_2019_4869_MOESM1_ESM.docx]

**Additional file 1: Behaviour specifications of the target behaviours.**

| **Behavioural specifications** | **Target Behaviour** | |
| --- | --- | --- |
|  | **Diet** | **Physical activity** |
| Who needs to perform behaviour? | All individuals with FH from the age of diagnosis, which can be any age from birth dependent on when individual is tested. Medical treatment begins at age 8-10 years but lifestyle advice can be followed from the age of 2 years. The physical activity guidelines referred to for this intervention to target are for those aged between 5 and 64 years of age, but younger and older individuals should also follow age appropriate guidance. This was not included in this intervention as individuals are expected to be aged between 10-64 years of age. | |
| What does person need to do differently to achieve desired change? | Choose to eat foods that enable them to meet the dietary intake guidelines (i.e. fruits, vegetables, wholegrains, plant stanols/sterols) and avoid eating too many foods that provide nutrients they need to limit (i.e. saturated fat and cholesterol) | Engage in physical activities and break up sedentary behaviours with periods of movement throughout the day. |
| When will they do it? | All eating or drinking occasions- every meal and snack through day and night | At some point everyday |
| Where will they do it? | In their own homes, places of education, workplaces, social events, restaurants and homes of friends or family members | In their own homes, places of education, workplaces, gyms and outside locations |
| How often will they do it? | For the majority of eating and drinking occasions- allowing for flexibility occasionally as not always possible/desired to choose the most suitable foods and drinks for their dietary needs. | A minimum of once a day for ≤ 18 years and a minimum of 3-5 times a week for >18 years.  Actions to break up sedentary behaviour to be carried out several times a day. |
| With whom will they do it? | Alone, with friends, family members and work colleagues | Alone, with friends, family members, work colleagues and sports teams. |
