# Supplementary material for: The development of a theory informed behaviour change intervention to improve adherence to dietary and physical activity treatment guidelines in individuals with familial hypercholesterolaemia (FH)
Source: BMC Health Serv Res. 2020 Jan 8;20:27. doi: 10.1186/s12913-019-4869-4 (PMC6950899; doi:10.1186/s12913-019-4869-4)
Supplement: Supplementary file 2 — Additional file 2. APEASE criteria evaluation of candidate intervention functions. [file 12913_2019_4869_MOESM2_ESM.docx]

**Additional file 2: APEASE criteria evaluation of candidate intervention functions.**

| **Intervention functions** | **Description** | **Canditate intervention function?** | **Does function meet the APEASE criteria in the context of adhering to lifelong dietary and physical activity guidelines and as part of an intervention within feasibility trial?** |
| --- | --- | --- | --- |
| Education | Increasing knowledge or understanding | Yes | Yes- intervention can include education about guidelines and also why they are important to follow. |
| Persuasion | Using communication to induce positive or negative feelsing or stimulate action | Yes | Yes- the benefits of following guidelines can be communicated during the intervention to induce positive feelings and inspire actions |
| Incentivisation | Creating an expectation of reward | Yes | No- providing rewards would not be sustainable for these behaviours which are to be adhered to for life and could not be continued after feasibility trial ended. Those who were only following guidelines to receive incentive would therefore be unlikely to continue with behaviours. |
| Coercion | Creating an expectation of punishment or cost | No | *Not applicable* |
| Training | Imparting skills | Yes | Yes- while it is not possible to include workshops to increase physical skills (i.e. cooking) within the intervention (due to affordability and practicality issues) individuals will be provided with information that will enable to development of skills and parents will be encouraged to impart skills to their children. |
| Restriction | Using rules to reduce the opportunity to engage in the target behaviour (or to increase the target behaviour by reducting the opportunity to engage in competing behaviours) | Yes | No- it is beyond the scope of the feasibility trial to change rules regarding the availability of foods or opporunities for physical activity. Furthermore, the results of the QES suggest that suggesting certain foods are restricted would only serve to increase the perceived difficultness of following the guidelines and would not be acceptable to the individuals. |
| Environmental restructuting | Changing the physical or social context | Yes | No- it is beyond the scope of the feasibility trial to change the physical or social contexts that individuals experience |
| Modelling | Providing an example for people to aspite to or imitate | No | *Not applicable* |
| Enablement | Increasing means/reducing barriers to increase capability or opportunity (beyond environmental restructuring) | Yes | Yes- the intervention can include strategies to enable individuals to feel more positively about the desired behaviours and the development of mental strength to engage in them. |

APEASE: Affordability, practicality, effectiveness/cost-effectiveness, acceptability, side effects, equity; QES: Qualitative evidence synthesis
