# Supplementary material for: The development of a theory informed behaviour change intervention to improve adherence to dietary and physical activity treatment guidelines in individuals with familial hypercholesterolaemia (FH)
Source: BMC Health Serv Res. 2020 Jan 8;20:27. doi: 10.1186/s12913-019-4869-4 (PMC6950899; doi:10.1186/s12913-019-4869-4)
Supplement: Supplementary file 3 — Additional file 3. Intervention checklist for 1st session [file 12913_2019_4869_MOESM3_ESM.doc]

**Additional file 3: Intervention checklist for 1^st^ session**

The following checklists provide an outline of topics to be covered in session 1 which is an hour-long face to face consultation with the dietitian which will be delivered to the parent-child dyad together.

The session is split into 8 sections, the aims of each are outlined in table below.

*Each participant is to be given an intervention booklet during this session which will be used during this session and for them to take home. It is to be handed out during section 3.*

| **Section** | **Aims for participants** |
| --- | --- |
| 1. Introduction and overview of intervention | -To understand the purpose of the intervention, what will be involved and what they will be asked to do |
| 2. Scientific rationale for intervention | -To understand the importance of nutrition and physical activity (PA)in the management of FH and for their overall health.  -To be aware of the importance placed on nutrition and PA by national and international guidelines for FH and the current recommendations in England that all FH patients should receive individualised advice about nutrition & PA  -To understand that the earlier treatment for FH starts, the more effective it is and this is why it is important to optimise nutrition and PA from a young age |
| 3. Education about nutrition targets | -To know what a healthy balanced diet looks like, including the food groups and the proportion each one should make to diet  -To know what the 5 nutrition targets of the intervention are  -To understand why each target is important for their health and for the management of their FH  -To understand what foods to include/exclude and/or increase/decrease consumption of to achieve targets |
| 4. Education about PA targets | -To know what they PA recommendations are for their age  -To know what the different levels of PA are (low, moderate and high) and what types of PA fall into which group  -To understand how they incorporate more PA into their lifestyle to help increase PA levels to recommended amounts (or more) |
| 5. Goal setting | -To agree with dietitian upon SMART goals for each of the targets. These will be changes to their lifestyle that they agree to make over the following 12 weeks to achieve nutritional intakes and PA levels closer to the targets. |
| 6. Barriers and solutions | -To identify potential barriers that may prevent them from meeting the goals set  -To identify, through discussion with dietitian and other family member, solutions to these barriers |
| 7. Follow up arrangements | -To understand what will happen over the following 12 weeks  -To understand the purpose of the weekly reflection diaries and know how to fill them out  -To know what will be discussed during these follow ups |
| 8. Wrap up | -An opportunity to ask any unanswered questions  -To receive summary of all that was discussed in session  -To receive encouragement and motivation from dietitian |

***Section 1: Introduction and overview of intervention***

|  | Completed |
| --- | --- |
| Welcome parent and child to the intervention part of the study |  |
| Explain the format of the intervention:   - This 1-hour meeting with me today - 4 follow up follow phone calls/ emails over the next 12 weeks - Explain what a dietitian is |  |
| Explain what will be involved in the intervention:   - A discussion about the nutrition and PA recommendations for people with FH and why they are important - Working together to agree upon some goals that each of you can make to help reach the nutrition and PA targets over the next 12 weeks - Identifying barriers to achieving these goals and working together to find solutions to these - Reviewing and adjusting the goals at each follow up session |  |
| Explain the approach of the intervention:   - This is not a restrictive, short term diet plan. It is advice to help you live a healthier lifestyle by providing you with up to date and correct information and helping you to decide upon what long term changes you can make - I’m not going to just tell you what you can and can’t eat. All the advice we discuss and goals we set together will be tailored to you and your needs and preferences - However, I would ask you to be open to trying new foods and activities as sometimes we need to do this to find out things we didn’t know we liked |  |

**Section 2: Scientific rationale for the intervention**

|  | Completed |
| --- | --- |
| Discuss the benefits treating FH (medication & lifestyle together):   - Unlike other causes of high cholesterol, as you have FH you need to take medication to help lower the amount of ‘bad’ LDL cholesterol - By lowering the amount of LDL cholesterol, you reduce your risk of developing heart problems when you are older, meaning you can live for longer and be in better health - We know that the best results are seen in those people who start their treatment at a young age, so even though you might feel healthy now it is important to follow the treatment your doctor gives you |  |
| Discuss where nutrition and PA fits into treatment for FH:   - Leading a healthy lifestyle (not smoking, being a healthy weight, eating well and being physically active) is **NOT** a replacement for medication - It can help directly by:   - Helping reduce your ‘bad’ LDL cholesterol even further than medication alone. This is achieved through eating more of certain foods that we know decrease LDL cholesterol and eating less of foods that we know increases LDL cholesterol   - Can increase your ‘good’ HDL cholesterol (medication can’t increase this)   - Delay how soon you will need to increase medication dose - It can also help manage your FH and it’s risks indirectly:   - Help manage other risk factors for heart problems such as blood pressure and weight   - Help you stay at a healthy weight which in turn can reduce the ‘bad’ LDL cholesterol - Research studies have found that among people with FH, the risk of developing a heart problem varies a lot. This research has found that the differences in the risk can be explained by the individual’s lifestyle. Those who are overweight or have high blood pressure are at higher risk   - This highlights the importance of living a healthy lifestyle |  |
| Discuss other benefits of living a healthy lifestyle:   - Being physically active can improve your mood and make you feel happier - Eating a nutritious diet can make you feel happier, have more energy, improve your skin and help you perform your best in school and work |  |
| Discuss the national and international guidelines for FH:   - NICE, a committee of experts who review all the available evidence from research studies and translate it into recommendations for doctors, recognise the importance of nutrition and PA in the management of FH - NICE state that all individuals with FH should receive advice from a dietitian to help them live a lifestyle that matches the nutrition and PA guidelines they recommend - Similar organisations across the world has also reviewed all the evidence and concluded upon the same nutrition and PA guidelines |  |
| Emphasis the benefits of living a healthy lifestyle:   - Even with medication, people with FH are still more likely to have heart problems earlier on than someone without FH - BUT there is evidence to show that for people with FH who start treatment early and live a healthy lifestyle, they can reduce this risk even further and expect to live as long as someone without FH |  |
| Discuss why we are having a family-based intervention:   - FH runs in families and it makes sense for you all to make changes together - Children learn lots of nutrition and PA habits from their parents, so by asking parents to lead healthy lifestyles this can help children - Parents often do lots of the cooking for younger children, so it is also important that they are on board with the lifestyle changes - Family members can offer support to each other which makes it easier - As the targets (apart from one which we will discuss) are the same as the healthy lifestyle advice given to people without FH, other members of your family can take part too and you can all help each other out |  |

**Section 3: Education about nutrition targets**

For each nutritional target discussion, there is listed content that should be communicated, however it is not to be just read out to participants. The information is to be delivered in a conversational style with child and their parent, encouraging them to be involved by asking them what they know, what they think etc. Try to get them to give the answers first.

|  | Completed |
| --- | --- |
| Give each participant an intervention booklet and explain that you will now go through what a healthy lifestyle looks like for people with FH- going through the nutritional and PA recommendations one by one. |  |
| Turn to page 2 and discuss the Eatwell plate,  Direct the following questions at the child but encourage participation/ help from parent:   - Have you seen the Eatwell plate before at school or elsewhere? - Would you be able to tell me a little about what it is? - Can you name one of the food groups? - What types of food do you think would be in this group?   Depending on how well they appear to understand the concept, discuss with them the Eatwell plate and what it represents:   - Represents what a healthy, balanced diet could look like. It shows how much food we eat should come from each food group - Explain each of food groups, including examples of common foods - Ask child what they ate for dinner last night and compare to the plate   Explain how this links to FH lifestyle recommendations   - The nutritional recommendations for FH are very similar to those recommended for people without FH - Everyone should use the eatwell plate as a guide - For FH management there are five really important things that we would like to pay particular attention to and that is what we will discuss next |  |
| Turn to page 3 and discuss target 1: Fats  Information to be communicated:   - Low fat diet is preferable for people with FH - Research evidence about SFA increasing LDL cholesterol - How to read labels to identify low fat foods - The difference between saturated and unsaturated fats - How to read labels to identify foods low in saturated fats - What foods are high in saturated fats - What foods are high in unsaturated fats - Food swaps to reduce saturated fats and increase unsaturated   Helpful prompts and questions:   - Do you know what saturated and unsaturated fats are? - What foods contain saturated fats? And unsaturated fats? - Do you eat any of these foods containing unsaturated fats? - Can you think of any swaps to reduce saturated fats? |  |
| Turn to page 4 and discuss target 2: Dietary cholesterol  Information to be communicated:   - The difference between blood and dietary cholesterol - Research about dietary cholesterol and LDL cholesterol in FH patients - Such small amounts in foods, doesn’t have impact upon blood cholesterol in most people. However, it can increase blood cholesterol levels in people with FH because they are not able to process cholesterol normally because of their faulty gene - Foods high in dietary cholesterol - How many of each you are able to eat per week   Helpful prompts and questions:  - Do you know what foods contain cholesterol?  - Why do you think that people with FH can’t eat too many of these? |  |
| Turn to page 5 and discuss target 3: eating more fruits and vegetables  Information to be communicated:   - At least 5 portions of fruit and vegetables per day - What a ‘portion’ is - High in vitamins, minerals and fibre - Tips for increasing amount of F&V into diets   Helpful prompts and questions:   - What is your favourite fruit/vegetable? - Why do you think fruits and vegetables are good for us? |  |
| Turn to page 6 and discuss target 4: eating more fibre  Information to be communicated:   - There are two types of fibre and both are important for staying healthy - However soluble fibre may help directly reduce LDL cholesterol by ‘trapping’ cholesterol found in other foods and stop it being absorbed - Foods high in soluble and insoluble fibre - Food swaps to increase fibre intake   Helpful prompts and questions:   - Do you know what the two types of fibre are? - Can you think of some foods that contain lots of fibre? |  |
| Turn to page 7 and discuss target 5: plant sterols/stanols  Information to be communicated:   - Plant stanols/sterols are found naturally in foods in small amounts. - They ‘compete’ with cholesterol and stop it being absorbed into the body which can reduce blood cholesterol - There is lots of research that it can reduce LDL levels of children and adults with FH even more than statins alone. - Easiest way to eat enough is to have a yogurt drink once per day - Must be taken every day and with main meal - Evidence to show they are safe in children aged 8 and above- but ONLY if have FH. Other children in family without FH should not have - Where they can be purchased and rough price estimate. Supermarket own brands 30p a bottle, brands about 60p a bottle. - Make sure to emphasis it is the cholesterol reducing yogurt drink, not the prebiotic/probiotic yogurt drinks   Helpful prompts and questions:   - Have you heard of plant sterols/stanols before? - Do you know what they do? - Have you tried one of these yogurt drinks before? |  |
| Allow for any questions from participants. |  |

**Section 4: Education about physical activity targets**

The information is to be delivered in a conversational style with child and their parent, encouraging them to be involved by asking them what they know, what they think etc. Try to get them to give the answers first)

|  | Completed |
| --- | --- |
| Explain that we are now going to discuss the PA recommendations for people with FH and ask to turn to page 8 |  |
| Discuss benefits of PA for general health:   - Lots of evidence to show people who are physically active have lower risk of heart disease, cancers and other diseases - PA can help you stay a healthy weight which reduces risk of developing any diseases - PA can improve your mood, energy levels and make you feel happier - It is also something you can do with your friends or family and bring you closer together   Discuss benefits of PA for FH management:   - People with FH are at higher risk of developing heart problems. As PA has been shown to reduce the risk of heart disease, it is extra important for people with FH to be physically active - In addition to the positives we already discussed, it can directly reduce your levels of LDL cholesterol and increase HDL cholesterol |  |
| Discuss the recommended levels of PA:  Information to be communicated:   - Recommended levels and types of PA for children and adults - What low, medium and high intensity means and examples of each - Importance of reducing sedentary time - That these are targets but no limit exists- the more PA and less sedentary time the better   Useful prompts and questions:   - Do you know how many minutes of physical activity it is recommended that you do? - What do you think might count as an activity that would be high intensity? - Do you play any sports? - What is your favourite activity to do during PE lessons at school? |  |

**Section 5: Goal setting**

|  | Completed |
| --- | --- |
| Encourage participant to set some specific goals for each of the nutritional targets (except for plant sterols/stanols) – best to be:   - Specific, measurable, attainable, realistic, time-based - E.g., Have fruit for dessert 3 evenings of the week OR switch to wholegrain bread for lunchtime sandwiches during weekdays when at school   Encourage discussion between parent and child to come to agreement upon these. For young children, the parents and childs goals may be the same whereas for older children who make some of their own food choices, they may have their own unique ones.  Ask participants to record their goals on pages 9 and 10.  ***[Please record goals in the dietetic intervention CRF]*** |  |
| Explain to participant that you would like them to use the ticklist on page 11 to record their intake of plant sterols/stanols drinks. Encourage dyad to:  -Stick up ticklist on the fridge and record daily if they consume the drink  -Agree upon a time that they will take it (ideally with main meal of day)  -Suggest a phone alarm reminder to be set |  |
| Encourage participant to set some specific goals for the PA targets. Best to be:   - Specific, measurable, attainable, realistic, time-based - E.g., walk to school 2 times per week instead of getting bus OR family walk every Saturday afternoon for 1 hour   Encourage participants to include some family based goals that they can do together for instance a family walk or cycle ride.  Ask participants to record their additional planned PA in the table on page 11  ***[Please record goals in the dietetic intervention CRF]*** |  |

**Section 6: Barriers and solutions**

|  | Completed |
| --- | --- |
| Discuss what barriers the participant thinks they could face when trying to achieve the goals they just wrote down. Encourage participant to record barriers in the ‘barriers and solution’ page in the intervention booklet. Parents and children encouraged to discuss together. |  |
| Discuss some solutions to these barriers, involving both parent and child:  *What do you think you could do to stop XX from getting in the way of eating more fruits and vegetables?*  Encourage participant to write down this solution in the ‘barriers and solution table’ in the intervention booklet.  ***[Please record barriers and solutions in the dietetic intervention CRF]*** |  |
| Ask participant to look back at this table when they are faced with these barriers over the upcoming 12 weeks. It may help to keep the table somewhere they see it regularly such as their bedroom or in the kitchen. |  |

**Section 7: Further support and follow up arrangements**

|  | Completed |
| --- | --- |
| Provide participant with links to suitable websites that can offer recipe suggestions and further details of the recommendations i.e. HEART UK website and NHS change4life |  |
| Provide participant with information about their local council organisations and fitness centres which they could access to help increase PA levels |  |
| Discuss with participants the importance of involving family and friends in your goals so that they will help hold you accountable |  |
| Discuss the importance of keeping track of your behaviour through self-monitoring and reflecting:   - Reflecting on our weeks helps us to be able to look back and see what we have done. It is easy to forget just what you have achieved when life is so busy - This is important, so you can celebrate your successes or see if you want to do a little bit more   Direct participants to the reflection diaries in the intervention booklet and explain that we would like them to use that space to record how they are getting on with the goals that they have set.  Suggest they both agree upon a time once a week that suits to do this.  Explain that these will be useful for our follow up conversations. |  |
| Agree with participants dates and times for the 2, 4, 8 and 11 week follow ups.  Obtain phone number from participant to call them on.  If they would prefer email contact then please take email address from them.  ***[Please record in dietetic intervention CRF and RC2 CRF]*** |  |

**Section 8: Wrap up**

|  | Completed |
| --- | --- |
| Thank participant for their participation over the last hour |  |
| Recap what was covered:  -What the nutritional and PA targets are  -Their goals for achieving those  -Their perceived barriers and solutions for these  -Reflection diaries  -Follow up phone call arrangements |  |
| Remind participant to contact anytime they would like outside of the follow ups if required |  |
| Explain to participant that we would like them to record their intakes and PA for 1 week like they did in the week before this meeting and agree upon the dates for recording and delivery of PA monitors  **[Please record in RC2 CRF]** |  |
| Thank participants again |  |
