# Supplementary material for: The development of a theory informed behaviour change intervention to improve adherence to dietary and physical activity treatment guidelines in individuals with familial hypercholesterolaemia (FH)
Source: BMC Health Serv Res. 2020 Jan 8;20:27. doi: 10.1186/s12913-019-4869-4 (PMC6950899; doi:10.1186/s12913-019-4869-4)
Supplement: Supplementary file 4 — Additional file 4. Intervention booklet for participants aged 14 years and above [file 12913_2019_4869_MOESM4_ESM.docx]

**Nutrition &**


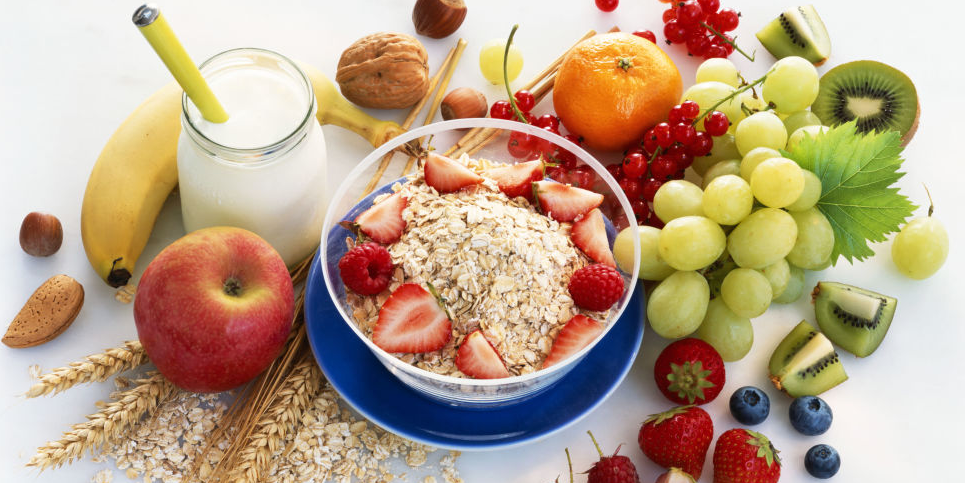
**Physical Activity
Plan**


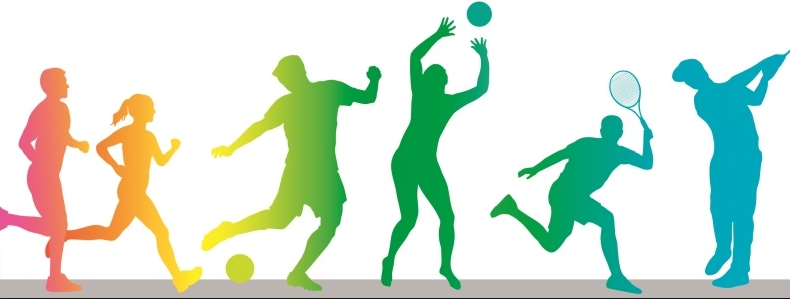


**Research study: Nutrition and Physical Activity Intervention for Families with Familial Hypercholesterolaemia (FH)**

**Name of participant: …………………………………………………………………………………………………**

If you have any questions then you can call or email the research dietitian XXXXXXXXXXX on:


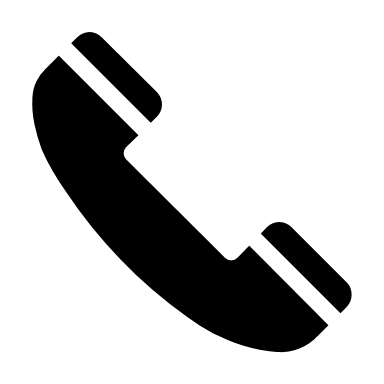
 XXXXXXXXX
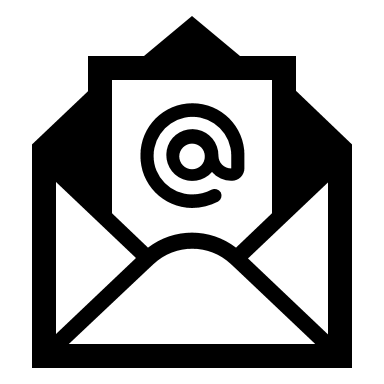
 XXXXXXXXXXX

**What does good nutrition look like?**

**
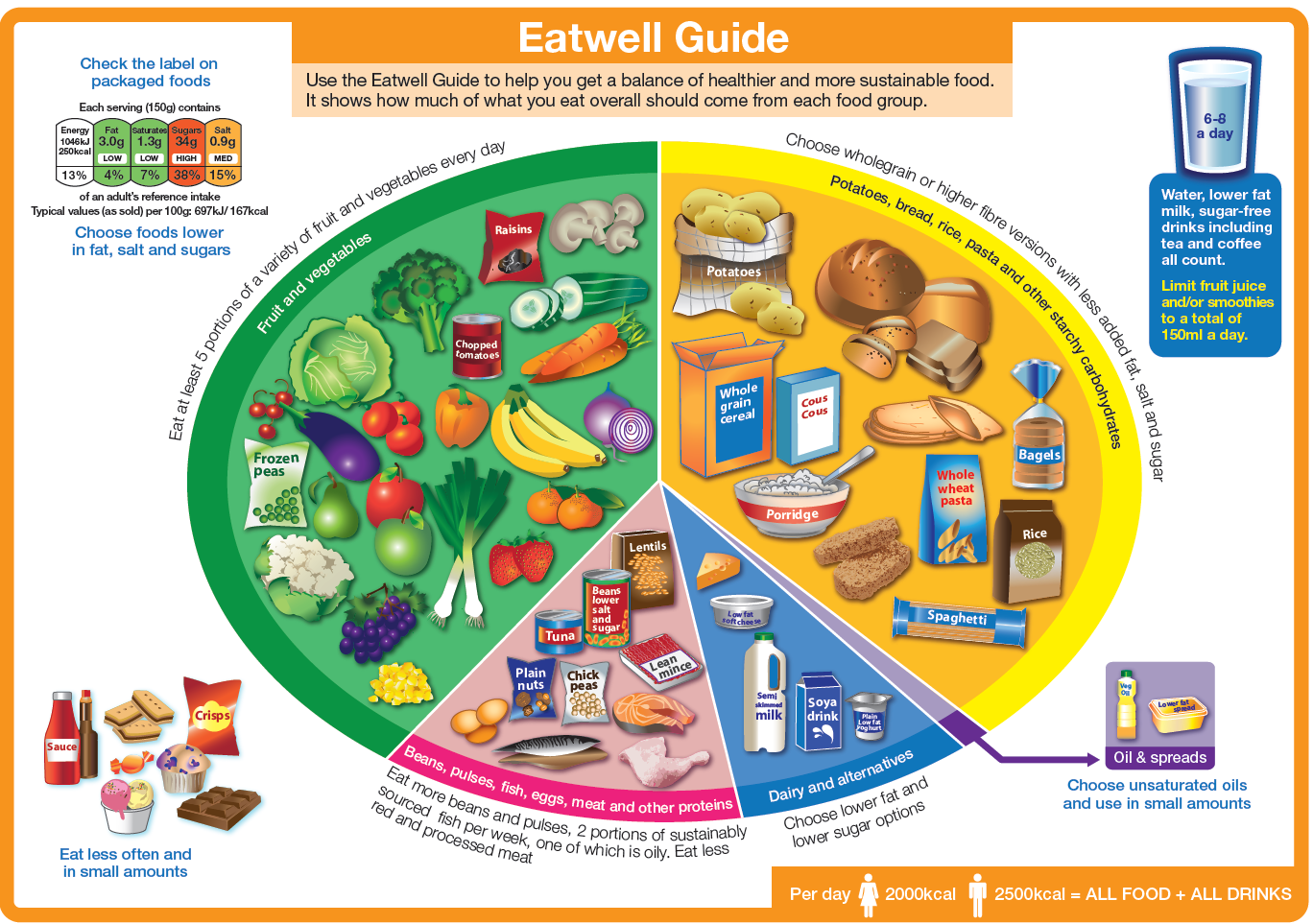
**

**Fats**

**How much fat should you eat?**


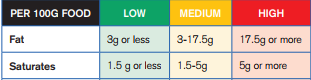
Everyone needs some fat in their diet- but it is important that you don’t have too much. Looking at labels on foods can help you choose foods low in fat. Looking at the **‘per 100g’** section of the label is the best option:

**What types of fat should you eat?**There are two different types of fat in the foods you eat- saturated and unsaturated. The saturated fats are the fats which can increase your blood cholesterol levels. You should try to eat fewer foods containing high levels of saturated fat and replace with foods rich in unsaturated fats.

**Foods containing saturated fats:
*Processed meats like sausages, fatty meats, whole milk and cream, hard cheeses, butter, lard, ghee, suet, palm and coconut oil.
Processed foods like cakes, biscuits, pastries and crisps***

**Foods containing unsaturated fats:
*Olive, sesame, soya, corn and rapeseed oils
Vegetable oil based margarines and spreads
Nuts, seeds, avocados
Oily fish like salmon, sardines and mackerel***

**Top tips:**

| **Eat less….** | **Swap it for …….** |
| --- | --- |
| Butter, ghee, lard, coconut and palm oil | Vegetable spreads and oils such as olive, rapeseed, sunflower and soya |
| Fatty meats and processed meats like sausages and bacon | Eat more chicken, turkey, fish or vegetarian options such as soya mince  Trim all visible fat from all meats  Have meat free days |
| Full fat milk, cheese, yogurt, cream and cream fraiche | Low fat options such as semi-skimmed or skimmed milk, low fat cheese and yogurts, soya alternatives |
| Cakes, crisps, chocolate, biscuits, pastries, pies or desserts with lots of cream or butter | Low fat yogurts, plain biscuits, fruit, low fat crisps, hot cross buns, nuts and seeds |
| Fried foods or foods roasted in butter or lard | Use vegetable oils for cooking or try methods such as steaming, boiling or grilling |
| Mayonnaise, creamy salad dressings and sauces like ranch dressing or cheese sauce for pasta | Use light mayonnaise, dressings made with olive or rapeseed oils, tomato based sauces for pasta |

**Target 1: Reduce how many foods high in saturated fat you eat and replace with foods rich in unsaturated fats**

**Dietary Cholesterol**

**Blood cholesterol and dietary cholesterol**

Did you know that most of the cholesterol in your blood is actually produced by your liver? It is needed for many processes in your body to keep you healthy but too much can cause heart disease. Small amounts of cholesterol are also found in some of the foods that you eat. This dietary cholesterol has only a small effect on your blood cholesterol levels. How much saturated fat you eat has more effect upon your blood cholesterol levels. However, as having FH causes your blood cholesterol levels to be higher than usual, it is important to make sure that you don’t eat too much dietary cholesterol.

**How much cholesterol should you eat?**

For people with FH, it is recommended that you eat less than 300mg of dietary cholesterol per day. By choosing foods low in saturated fat and eating a healthy balanced diet, it is unlikely you will eat more than this. However, there are some foods that are low in saturated fat but contain a large amount of dietary cholesterol. If you normally eat lots of these foods then you could be having too much dietary cholesterol.

**What foods contain cholesterol?**

All foods from animals (meat and dairy) contain some cholesterol. By choosing foods low in saturated fat you will avoid eating too much cholesterol from these sources.


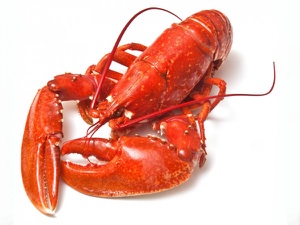
However, there are a few foods that are low in saturated fat but high in dietary cholesterol:

**Organ meats**

***Liver, kidney, heart, tripe & sweetbreads***

**Egg yolks**

**1 yolk= 180mg**

**Shellfish**

***Prawns, lobster, crab, squid & octopus***


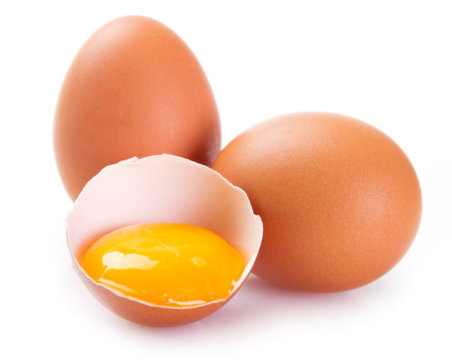

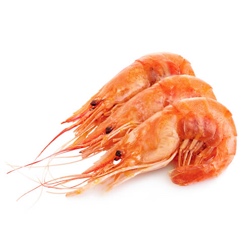

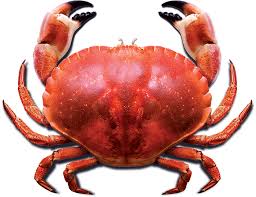

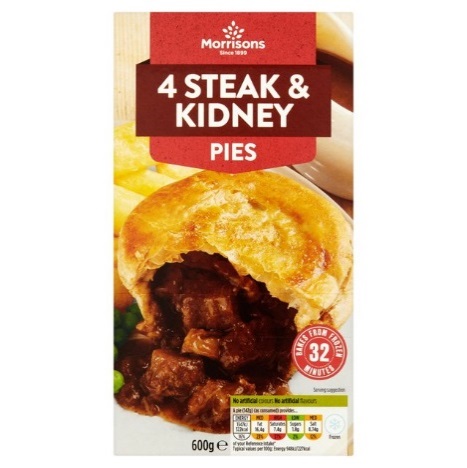

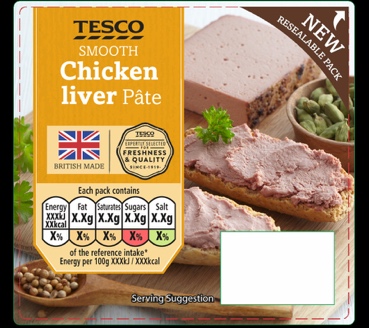
 **Top tips:
1.Follow the top tips for reducing saturated fat intake
2. Eat organ meats or shellfish no more than once a week
3. Eat no more than 3-4 eggs per week**

**Target 2: Limit how many foods high in dietary cholesterol you eat per week**

**Fruits and vegetables**

Fruits and vegetables are high in vitamins, minerals and fibre. You should be trying to eat AT LEAST 5 portions of fruits and vegetables each day- the more the better! Try to eat of variety of different types every day to help increase the variety of vitamins and minerals you get. **Fresh, frozen, canned and dried- they all count!**

**1 portion counts as:**


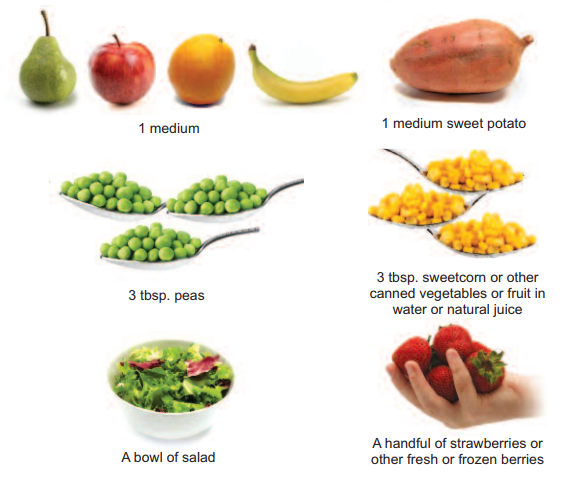


**Top tips for fitting in more fruit and vegetables:**

| **Breakfast** | **Lunch** | **Dinner** | **Snacks** |
| --- | --- | --- | --- |
| Top cereal with dried fruit or berries | Add salad to your sandwich or wrap | Always serve meals with salad or cooked vegetables | Fresh fruit |
| Have a banana with a cereal bar on the go | Vegetable soups | Bulk out stews, casseroles and curries with vegetables | Dip vegetables into hummus or salsa or guacamole |
| A small glass of fruit juice | Add fruit to yogurt | Add vegetables into pasta sauces or into side dishes like potatoes or rice | Sugar free jelly or low fat custard with fruit |

**Target 3: Increase the number of portions of fruits and vegetables you eat each day**

**Fibre**

| **Soluble fibre rich food groups** | **What foods can I find this in?** | **Ideas for how to add it into your daily diet** |
| --- | --- | --- |
| **Oats** | Porridge oats Oat based cereal i.e. oatabix  Oatcakes | Bowl of porridge or oat based breakfast cereal Oatcakes topped with low fat cream cheese  Homemade flapjacks made with oats |
| **Beans** | Baked, kidney, butter, haricots, cannellini, flageolet, pinto, broad & borlotti beans | Baked beans on toast or a baked potato  Use beans in meals such as chilli, fajitas & curries  Add to casseroles and soups |
| **Lentils** | Green, red, yellow and brown | Add to soups Use in place of meat in curries and casseroles |
| **Peas** | Garden and sugar-snap | Frozen peas are a great stand-by to have to serve with dinners or add to pasta or rice dishes  Snack on raw sugar-snap peas- try with a low fat dip like salsa |
| **Fruits and vegetables** | Broccoli, sweet potato, aubergine, apple, strawberries and prunes are particularly rich sources | Have baked sweet potato or oven cooked chips Add prunes to your cereal, porridge or yogurt Snack on apples and strawberries |
| **Nuts and seeds** | Almonds, peanuts, cashews, pecans, flaxseeds, chai seeds | Sprinkle tablespoon of chai seeds or flaxseeds on cereal/porridge  A handful of nuts makes a great portable snack |

Fibre is a really important nutrient which can help lower blood cholesterol. Good sources include fruits, vegetables, pulses, beans and wholegrain varieties of bread, pasta, noodles and breakfast cereals. There are two different types of fibre: soluble and insoluble. Both are important for your health and blood cholesterol levels, but soluble fibre in particular has been found to lower blood cholesterol.
**Top tips to increase soluble fibre intake:**

**Top tips to increase insoluble fibre intake:**

| **Insoluble fibre rich foods** | **Top tips** |
| --- | --- |
| **All fruits and vegetables** | Keep the skin on fruits and vegetables when possible |
| **Wholegrains** | Choose brown pasta, bread, rice & noodles (Ensure it says ‘wholegrain’ in ingredients) If the family only like white bread- try the ‘50/50’ versions first Choose wholegrain versions of breakfast cereals |
| **Popcorn** | Pop kernels yourself or choose plain versions. Avoid sweet or buttery options |
| **Dried fruit** | Naturally sweet, a small handful of dried fruit can satisfy your sweet tooth |

**Target 4: Increase how many insoluble and soluble fibre-rich foods you eat each day**


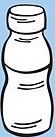
**Plant stanol or sterols**

***What are they?***

Plant stanols and sterols are found naturally in plants. They have been found to lower blood cholesterol levels.

***How do they work?***

They compete with cholesterol for absorption which means less cholesterol is absorbed into the body from the gut. The body in turn removes more cholesterol from the blood, to make up from the smaller amount it gets from the gut. This results in lower blood cholesterol levels.

***Are they safe?***

They have been extensively researched and approved for use in adults and children with FH. Some children may experience a slight decrease in the levels of some micronutrients in their blood. However, this can be avoided by eating plenty of fruits and vegetables. They are not recommended for children who don’t have FH, so please make sure they do not drink them.

***How much should you have?***

The research suggests you have between 1.5g and 2.4g each day, with no benefit of having more than this. Most importantly- they must be taken every day, and alongside meals, to have the cholesterol lowering effect.


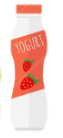
***What foods are they in?***

The easiest way to make sure you consume the recommended amount per day is to have 1 fortified mini yoghurt drink per day. There is a range of branded and own brand (e.g. supermarket own brand) options. They are found in the chilled sections of large supermarkets. There are many different flavours to try.

***How expensive are they?***The own brand options cost approximately 30p per bottle. The branded options are between 50-60p per bottle.

**Target 5: Have 1 plant stanol or sterol yoghurt drink every day**

**Physical activity**

***What are the benefits?***

Being physically active is important for everyone. There is lots of research to show that it reduces the risk of diseases such as coronary heart disease. For people with FH, who have a higher risk of developing coronary heart disease, these benefits are especially important. Physical activity may reduce LDL cholesterol and increase HDL cholesterol- which is the ‘good’ type of cholesterol.

***How much do you need to do?***

Adults should be aiming to fit in 150 minutes of moderate activity each week. This can be split into **small 10 minute bouts** spread across the week. Alternatively, you can aim to achieve 75 minutes of vigorous intensity exercise. Or you could have a combination of both levels of intensity.

Children and young adults should be aiming for at least 60 minutes of moderate to vigorous activity on every day of the week. On 3 of these days the activity should be vigorous intensity.

For adults and children, it is important to reduce the amount of time you spend sitting down. Research has found this to be associated with bad health outcomes even if you manage to meet the above recommendations!

***These recommendations are the MINIMUM amount- the more physical activity you can manage the better!***

**What counts?**

**Moderate intensity** physical activity = you feel warmer and breath harder, but you can still hold a conversation

**Fast walking Cycling Gardening Housework Walking the dog Dancing**

**Vigorous intensity** physical activity = you feel warmer, breath much harder, heart beats fast and you would find it difficult to speak to someone

**Running Swimming Football Netball Rugby Hockey Walking fast uphill**

**How can I do more?**

**My targets and goals**

| **My agreed goals** | **Any changes at weeks 2, 4, 8 or 11?** |
| --- | --- |
|  |  |
|  |  |
|  |  |

**Target 1: Reduce saturated fats and increase unsaturated fats**

**Target 2: Limit how many foods high in dietary cholesterol you eat each week**

| **My agreed goals** | **Any changes at weeks 2, 4, 8 or 11?** |
| --- | --- |
|  |  |
|  |  |
|  |  |

**Target 3: Increase the number of portions of fruits and vegetables you eat each day**

| **My agreed goals** | **Any changes at weeks 2, 4, 8 or 11?** |
| --- | --- |
|  |  |
|  |  |
|  |  |

**Target 4: Increase how many insoluble and soluble fibre-rich foods you eat each day**

| **My agreed goals** | **Any changes at weeks 2, 4, 8 or 11?** |
| --- | --- |
|  |  |
|  |  |
|  |  |

**Target 5: 1 Plant stanol or sterol yoghurt each day**A reminder to stick on fridge**:** Tick off each day after you have your plant stanol or sterol drink!
Please remember to bring in to the research dietitian at the end of the 12 weeks.

**Remember it is best to have it with your evening meal! It is also important to have EVERY day for most benefit!**

| **Week** | **Monday** | **Tuesday** | **Wednesday** | **Thursday** | **Friday** | **Saturday** | **Sunday** |
| --- | --- | --- | --- | --- | --- | --- | --- |
| **1** |  |  |  |  |  |  |  |
| **2** |  |  |  |  |  | 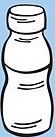 |  |
| **3** |  |  |  |  |  |  |  |
| **4** |  |  |  |  |  |  |  |
| **5** |  |  |  |  |  |  |  |
| **6** |  |  |  |  |  |  |  |
| **7** |  |  |  |  |  |  |  |
| **8** |  |  |  |  |  |  |  |
| **9** | 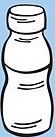 |  |  |  |  |  |  |
| **10** |  |  |  |  |  |  |  |
| **11** |  |  |  |  |  |  |  |
| **12** |  |  |  |  |  |  |  |

**Target 6: Increasing how physically active you are and reducing sedentary time**

| **Day** | **What I currently do** | **What I will add in** |
| --- | --- | --- |
| **Monday** |  |  |
| **Tuesday** |  |  |
| **Wednesday** |  |  |
| **Thursday** |  |  |
| **Friday** |  |  |
| **Saturday** |  |  |
| **Sunday** |  |  |

**Barriers and solutions worksheet**

| **Barrier** | **Possible solution(s)** |
| --- | --- |
|  |  |
|  |  |
|  |  |
|  |  |
|  |  |
|  |  |
|  |  |
|  |  |

**Reflection diaries (To be completed each week)
Use the space below to reflect on how you have been getting on with making the agreed changes you agreed with the dietitian in this booklet. It might be easiest to schedule in a time each week to do this with your parent/or child. You can use these notes to discuss progress with dietitian during the follow up phone calls or emails. There is one diary for each week. The plant sterol/stanol target progress will be recorded on the ticklist!
Week 1 Reflection diary**

| **Targets** | **What I have done well this week?** | **What did I find difficult this week?** | **Any questions for dietitian?** |
| --- | --- | --- | --- |
| **Reducing saturated fat** |  |  |  |
| **Reducing foods high in cholesterol** |  |  |  |
| **Increasing fruits and vegetables** |  |  |  |
| **Increasing fibre rich foods** |  |  |  |
| **Increasing physical activity** |  |  |  |

**2 week check in Phonecall:**

………………...........................................................................................................................

**4 week check-in phonecall:**
………………...........................................................................................................................

**8 week check-in phonecall:**………………...........................................................................................................................

**11 week check-in phonecall:**………………...........................................................................................................................

**End of study clinic visit (remember you and your child must be fasted for at least 6 hours)**

…………………………………………………………………………………………………………

**Recording everything you eat and drink for 4 days between:**
………………………………………............................................................................................

**Recording physical activity for 7 days between:**

…………………………………………………………………………………………………………

**Get in touch:**

If you have any questions then you can call or email the research dietitian xxxxxxxxxxxxxxxx on:


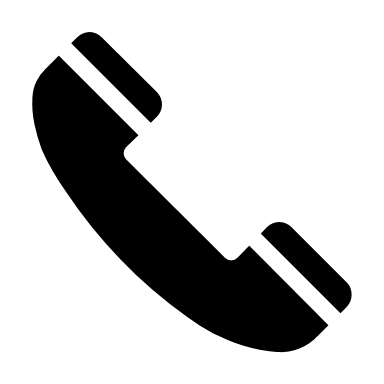
 xxxxxxxxxxxxxxxxx
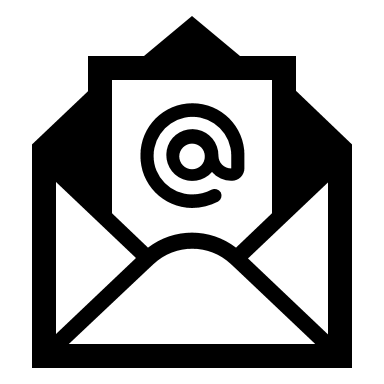
 xxxxxxxxxxx

Remember to check out the following websites for more useful tips:
[www.heartuk.org.uk/cholesterol-and-diet](http://www.heartuk.org.uk/cholesterol-and-diet)
[www.nhs.uk/change4lifE](http://www.nhs.uk/change4lifE)
[www.nhs.uk/oneyou/active10](http://www.nhs.uk/oneyou/active10)
